# Enhanced multicancer screening assay through whole-genome methylation sequencing-based multimodal cell-free DNA analysis

**DOI:** 10.1038/s12276-026-01674-7

**Published:** 2026-04-21

**Authors:** Seongmun Jeong, Dayoung Go, Yujin Jeon, Yong-Jin Kim, Hayoon Lee, Young-Woo Kim, Hyun Goo Woo, Joo Kyung Park, Young Tae Kim, Young Sik Park, Se-Hoon Lee, Wonshik Han, Han-Byoel Lee, Kwang Hyun Kim, Sanghoo Lee, Sungho Kim, Sang-Hyun Song, Hwang-Phill Kim, Yongjun Cha, Duhee Bang, Tae-You Kim

**Affiliations:** 1 IMBdx Inc., Seoul, Republic of Korea; 2 Department of Cancer Policy and Population Health, National Cancer Center Graduate School of Cancer Science and Policy and Center for Gastric Cancer and Department of Surgery, National Cancer Center, Goyang, Republic of Korea; 3 Department of Physiology, Ajou University School of Medicine, Suwon, Republic of Korea; 4 Department of Medicine, Samsung Medical Center, Sungkyunkwan University School of Medicine, Seoul, Republic of Korea; 5 Department of Thoracic and Cardiovascular Surgery, Seoul National University College of Medicine, Seoul National University Hospital, Seoul, Republic of Korea; 6 Division of Pulmonary and Critical Care Medicine, Department of Internal Medicine, Seoul National University Hospital, Seoul, Republic of Korea; 7 Division of Hematology-Oncology, Department of Medicine, Samsung Medical Center, Sungkyunkwan University School of Medicine, Seoul, Republic of Korea; 8 Department of Health Sciences and Technology, Samsung Advanced Institute of Health Sciences and Technology, Sungkyunkwan University, Seoul, Republic of Korea; 9 Department of Surgery, Seoul National University College of Medicine, Seoul, Republic of Korea; 10 Department of Urology, Ewha Womans University Seoul Hospital, Seoul, Republic of Korea; 11 Seoul Clinical Laboratories Healthcare Inc., Yongin-si, Republic of Korea; 12 SUNY Upstate Medical University, Syracuse, NY USA; 13 Department of Chemistry, Yonsei University, Seoul, Republic of Korea; 14 Cancer Research Institute, Seoul National University, Seoul, Republic of Korea; 15 Department of Molecular Medicine and Biopharmaceutical Sciences, Graduate School of Convergence Science and Technology, Seoul National University, Seoul, Republic of Korea

**Keywords:** Cancer screening, Diagnostic markers

## Abstract

The rapid and accurate detection of multiple cancers presents considerable challenges, especially for stage I disease, due to the low concentration and heterogeneous nature of circulating tumor DNA. Here we introduce an enhanced multicancer screening assay that integrates whole-genome methylation sequencing with an innovative multimodal analytical framework for cell-free DNA. The ensemble machine learning model integrates four specific cell-free DNA characteristics: average methylation fraction, copy number variation, fragment size ratio and fragment size distribution. The model underwent testing on 1415 samples, encompassing eight primary cancer types and healthy controls. The sensitivity was 93.2%, and the specificity was 95%. The test demonstrated effectiveness in detecting cancers at early stages. The sensitivity was 92.3% for stage I and 92.2% for stage II. The multimodal technique successfully combined average methylation fraction’s sensitivity to early epigenetic signals with fragmentomic characteristics. This facilitated the differentiation between healthy individuals and those with early stage cancer. The model achieved an accuracy rate of 85.7% in the top 2 category for correctly identifying the tissue of origin. The results confirm that whole-genome methylation sequencing-based multimodal analysis can improve multicancer early detection technology and revolutionize cancer screening methods.

## High-sensitivity multicancer test targets early disease stages

Cancer is a leading cause of death worldwide, with millions of new cases each year. Early detection is crucial for improving survival rates, but current screening methods can be invasive and expensive. This study explores a new approach using a blood test called liquid biopsy. This test looks for cell-free DNA (cfDNA) in the blood, which can show signs of cancer. Researchers developed a method called CancerFind, which uses machine learning to analyze cfDNA for cancer detection. They tested this on 1,048 patients with cancer and 387 healthy individuals. The study focused on eight types of cancer, including breast and lung cancer. The method combines different features of cfDNA, such as methylation patterns and DNA fragment sizes, to improve detection accuracy. The results showed that CancerFind could detect cancer with high sensitivity and specificity, even in early stages.

This summary was initially drafted using artificial intelligence, then revised and fact-checked by the author.

## Introduction

Cancer continues to be a primary cause of mortality globally, with more than 20 million new cases and nearly 9.7 million deaths reported annually^1,2^. Early detection is essential for enhancing survival rates and optimizing treatment outcomes, as prompt intervention facilitates curative treatment^3^ and mitigates the challenges associated with late-stage diagnosis. Conventional screening methods, including imaging and tissue biopsy, are often invasive, costly and limited in their capacity to concurrently detect multiple cancer types, despite the significance of early detection^4^. In addition, numerous high-mortality cancers, such as pancreatic, ovarian and prostate cancers, do not have established screening guidelines, leading to late-stage diagnoses and unfavorable prognoses^5^. Furthermore, it has been demonstrated that MCED tests can detect a broad range ofearly-stage cancers that lack USPSTF-recommended screening protocols^6^. These limitations highlight the pressing necessity for noninvasive, cost-effective and comprehensive strategies for multicancer early detection (MCED).

Liquid biopsy represents a significant advancement in noninvasive cancer screening methodologies^7,8^. Cell-free DNA (cfDNA) has garnered considerable interest because it effectively reflects the genetic and epigenetic signatures associated with tumor biology^9,10^. Circulating tumor DNA (ctDNA) originating from tumor cells offers essential insights into cancer detection. Recent developments in next-generation sequencing (NGS) and bioinformatics have facilitated the thorough characterization of cfDNA attributes, encompassing DNA methylation patterns, copy number variations (CNVs) and fragmentomic features^11,12^. DNA methylation profiling exhibits remarkable specificity for both tissue and cancer types, establishing it as a fundamental component that demonstrates exceptional tissue- and cancer-type specificity, making it a cornerstone of many MCED strategies^13,14^.

Multiple cfDNA-based MCED methodologies have shown feasibility for cancer detection, with differing degrees of sensitivity and specificity. The Galleri test, developed by GRAIL, uses targeted methylation profiling to identify more than 50 cancer types, achieving a specificity of 99.5% and a sensitivity of 51.5%^15–17^. DELFI’s fragmentomics technology uses advanced machine learning algorithms to analyze cfDNA fragmentation patterns throughout the genome, attaining a sensitivity exceeding 90% for lung cancer detection with a specificity of 50%^18–20^. Similarly, CancerSEEK integrates genetic mutations and protein biomarkers to identify eight cancer types, exhibiting sensitivities ranging from 33% to 98%, with a specificity of approximately 99%^14,21,22^. Despite advancements, current methods encounter difficulties in optimizing sensitivity, especially for early stage cancers, and ensuring broad applicability across various patient populations.

We have previously established a cfDNA analysis methodology for early cancer detection that integrates whole-genome methylation profiling, average methylation fraction (AMF) with CNV analysis and fragmentomic signatures^23^. Using whole-genome methylation sequencing, we conducted a comprehensive analysis of genome-scale methylation to capitalize on the discernible benefits of other verifiable cancer-related variations. We developed the CancerFind test, which is a multifeature cancer signature ensemble (CSE) classifier that surpasses individual feature classifiers. CancerFind demonstrated 95.2% specificity and 88.9% sensitivity, indicating that epigenetic and genomic data can facilitate early cancer detection^23^.

A key challenge in cfDNA-based MCED is the low abundance of ctDNA relative to nontumor-derived fragments^24,25^. As most cfDNA originates from normal cells with distinct methylation and fragmentation patterns, tumor-specific signals can be obscured by this high background noise^26–28^. Consequently, novel analytical methods capable of distinguishing subtle tumor-derived signals from the overwhelming presence of nontumor-derived cfDNA are urgently required.

To address these limitations and improve early cancer detection across multiple cancer types, we developed a multimodal MCED framework that integrates multiple cfDNA features, including AMF for methylation profiling, CNV analysis for genomic alterations and fragmentomics analysis (fragment size ratio (FSR) and fragment size distribution (FSD)). Each feature captures unique tumor-specific signals, providing complementary insights into ctDNA biology. AMF quantifies global methylation levels in specific regions^29–31^. CNV analysis identifies genomic amplifications or deletions that are linked to cancer progression. FSR and FSD utilize variations in cfDNA fragmentation profiles between tumor-derived and normal fragments to improve the sensitivity of cancers characterized by low ctDNA abundance.

This study sought to identify the limitations of current MCED methods and to emphasize the distinctive contributions of our research. This study assessed the efficacy of a multimodal framework in identifying eight prevalent cancer types at all stages of progression: colorectal, gastric, liver, pancreatic, lung, breast, ovarian and prostate. Our approach utilizes an ensemble machine learning model that incorporates AMF, CNV, FSR and FSD features to enhance sensitivity while preserving high specificity for early stage cancers. We also evaluated the capacity of the model to predict the tissue of origin (TOO) with high accuracy. These findings illustrate the substantial potential of combining various cfDNA characteristics within a unified diagnostic framework to enhance noninvasive cancer screening.

## Materials and methods

### Study design and sample collection

This study used a retrospective cohort of patients diagnosed with eight cancer types—colorectal, gastric, liver, pancreatic, lung, breast, ovarian and prostate—along with a cohort of healthy individuals. Participants were recruited following the approval form of the institutional review boards of multiple hospitals. The eligibility criteria for patients with cancer require histological confirmation of the disease type and stage according to the eighth edition of the American Joint Committee on Cancer guidelines^32^. The healthy control group was composed of individuals without a history of cancer or other major diseases. Plasma samples from patients with cancer were acquired by separating and preserving the blood drawn before the commencement of treatment, whereas whole blood samples were collected from healthy subjects.

### Sample processing and cfDNA extraction

Plasma was isolated from whole blood by centrifugation at 1500*g* for 15 min, followed by an additional centrifugation step at 16,000*g* for 10 min to remove cellular debris. cfDNA was extracted from plasma using the Maxwell RSC ccfDNA Plasma Kit (Promega) following the manufacturer’s protocol. DNA concentration and quality were assessed using the cfDNA ScreenTape Assay on an Agilent 4200 TapeStation system. For genomic DNA, tumors and matched normal tissue samples were extracted using the Maxwell RSC Tissue DNA Kit (Promega) and quantified using the Qubit double-stranded DNA High Sensitivity Kit (Thermo Fisher Scientific).

### Library preparation and sequencing

A methylation-sequencing library was constructed using the IMBdx AlphaLiquid screening platform. The library underwent enzymatic conversion and was sequenced on the NovaSeq 6000 platform (Illumina), yielding approximately 100 Gb of raw data per sample with 150 bp paired-end reads.

### NGS data preprocessing

Raw sequencing reads were converted to FASTQ format using the Illumina bcl2fastq software (v2.20.0.422) and subsequently processed with fastp^33^ (v0.23.1) to eliminate adapter sequences and low-quality reads. The reads were aligned to the GRCh37 human reference genome using bitmapperBS^34^ (v1.0.2.1) and subsequently processed into BAM files using SAMtools^35^ (v1.14). PCR duplicates were identified and eliminated using the MarkDuplicates function of GATK (v4.2.3.0)^36^. The quality control metrics included a mapping rate exceeding 80%, duplication rate below 25% and methylation conversion efficiency exceeding 99%.

### AMF and DMRs

To identify cancer-specific methylation markers, we analyzed 28,245,162 CpG dinucleotide sites across the genome using the GRCh37 human reference genome. CpG sites with less than 3× coverage in healthy individuals were excluded from the analysis. The methylation status was classified into three categories: unmethylated (bottom 10% methylation level, *β* < 0.5), methylated (top 10% methylation level, *β* > 0.5) and neutral (remaining sites). Contiguous CpG sites with the same methylation status were grouped into regions and further refined to improve their biological relevance and computational efficiency. CpG sites located on sex chromosomes or within regions flagged on the ENCODE blacklist were excluded^37^. The methylation levels in each region were quantified using AMF.

$${\mathrm{AMF}}_{i}=\frac{1}{\left|{R}_{i}\right|}{\sum }_{j\in {R}_{i}}\frac{{C}_{j}}{{C}_{j}+{T}_{j}},$$

fwhere ${C}_{j}$ and ${T}_{j}$ represent cytosine and thymine count at the *j*th CpG site, respectively, and where ${R}_{i}$ denotes all CpG sites within region $i$.

Differentially methylated regions (DMRs) were identified using *t*-tests across three comparison settings: (1) tumor tissue versus adjacent normal tissue, (2) specific cancer type versus other cancer types and (3) specific cancer type versus all other cancers combined. A false discovery rate (FDR) threshold of 0.05 was applied to select statistically significant DMRs. The concordance between cfDNA-derived markers and tissue-derived markers was used to finalize DMR selection.

DMRs were identified in colorectal, liver and lung cancers by comparing cancerous tissues with neighboring normal tissues (Supplementary Fig. 1). For cancer types lacking matched normal tissue, cancer-specific markers were identified by evaluating the statistical significance of differences in cfDNA methylation between patients with cancer and healthy individuals using *t*-tests with a FDR threshold of 0.05 (Supplementary Fig. 2). Marker selection was further refined on the basis of concordance with cfDNA-derived signals. This concordance was assessed via the same statistical tests on cfDNA from healthy individuals and patients with cancer within the training set, with the final markers selected on the basis of their directional consistency with tissue-derived markers (Supplementary Fig. 3). The selected markers corresponding to patients with and without normal tissues are summarized in Supplementary Tables 2 and 3, respectively.

To address the issue that tissue-based markers are not well represented in cfDNA owing to tumor heterogeneity, we further selected and refined cfDNA-based markers. Markers exhibiting consistent trends in both tissue and cfDNA were filtered, resulting in the selection of 407,142 and 724,607 markers for constructing cancer signal detection (CSD) and TOO prediction datasets, respectively (Supplementary Tables 2 and 3). A comparison of unsupervised clustering before and after the selection of CSD and TOO markers indicated that the delineation of clusters among healthy individuals became more pronounced after selection, signifying an enhancement in classification efficacy.

Supplementary Table 4 presents how this strict refinement process affects assay performance in a measurable way. This table shows how the AMF single-feature models’ diagnostic performance changes before and after these steps for selecting cfDNA-specific markers and checking for agreement. This comparison clearly shows that adding cfDNA-specific signals, guided by quantitative concordance, improves detection performance, especially for cancers that are still in their early stages.

### CNVs

CNVs were analyzed using CNVkit software, which detects genomic regions with copy number gains or losses relative to a reference derived from healthy controls^38^. The quantitative features included the fraction of copy number calls within predefined bins.

To improve the detection accuracy for early stage cancers with low CNV burdens, and critically, to mitigate the risk of false positives arising from age-related, nonmalignant somatic changes such as clonal hematopoiesis of indeterminate potential, we systematically excluded genomic regions commonly altered in healthy individuals from our analysis.

The specific exclusion criteria were defined as follows: any genomic region (for example, a specific cytoband or $\beta \mathrm{-}\mathrm{Mb}$ region) where a specific CNV marker (for example, a copy number alteration exceeding 2*σ*) was detected in more than *α*% (for example, 5% or 10%) of the healthy control cohort was excluded from the final CNV marker set.

To provide the quantitative validation of the benefit derived from applying these exclusion criteria, we have included Supplementary Table 4. This Supplementary Table presents a comparison of the sensitivity within the healthy control cohort before and after the application of the CNV exclusion criteria, clearly demonstrating that this crucial filtering step improved the model’s sensitivity and clinical reliability by effectively eliminating nonmalignant signals.

The CSD score was calculated by identifying regions where gain markers satisfied the conditions of healthy <*α*% and cancer >*β*%, respectively. (Supplementary Fig. 6). The frequencies at which these markers were detected in each sample were used in the calculation. Marker selection and optimization of the *α*% and *β*% thresholds were conducted within the training set. The model for calculating the TOO prediction score was built using TensorFlow’s^39^ Keras library.

### FSD

FSD patterns were analyzed by binning cfDNA fragment lengths into 5-bp intervals within the range of 80–220 bp using SAMtools software^35^. Tumor-derived cfDNA fragments are typically shorter than those derived from healthy cells, which results in distinct fragmentation profiles. The FSD features were used to construct a multiclass classification model using support vector classification^40^. Data standardization was performed using the standard scaler^41^.

### FSR

FSR was examined as a critical fragmentomic characteristic to distinguish tumor-derived cfDNA fragments from those derived from normal cells. cfDNA fragments were categorized into specific size intervals, with emphasis on short fragments (>80 and <150 bp) and long fragments (>150 and <220 bp). The FSR was determined by calculating the ratio of short to long fragments within the cell-free DNA (cfDNA) pool. This metric utilizes the observation that tumor-derived cfDNA fragments are primarily shorter owing to fragmentation patterns driven by apoptosis. Fragment length extraction and subsequent binning were conducted using SAMtools^35^ for FSR analysis. The ratios were standardized across samples to address variations in the total cfDNA yield. The FSR data were incorporated into the ensemble machine learning framework to improve the sensitivity of CSD, particularly for cancers characterized by low ctDNA abundance.

### Single-feature model development

For each feature (AMF, CNV, FSD and FSR), the optimal model was identified on the basis of the AUC using the best_model function from the PyCaret Python package (https://github.com/pycaret/pycaret). This function encompasses algorithms such as support vector machine, random forest^42^, gradient-boosting machine^43^ and logistic regression. Hyperparameter optimization was conducted within the PyCaret framework utilizing fourfold cross-validation to ensure rigorous model evaluation. Each single-feature model was trained to classify the samples into nine distinct categories: one for the healthy controls and one for each of the eight cancer types. CSD was established by comparing the scores of healthy individuals with the cumulative scores of the eight cancer types for the other three features, excluding CNV, with a threshold set to achieve 95% specificity. The efficacy of the TOO prediction model for the four features was assessed using the top 1 and top 2 selection approaches, which depend on conditional probabilities and omit the scores of healthy individuals in the nine-class classification.

### Ensemble model development

We combined single-feature models into an ensemble framework to further enhance diagnostic robustness and accuracy. In this method, the estimated sample-specific cancer probabilities of each single-feature model are first normalized using a logit transformation. After accounting for the effects of AMF, CNV, FSR and FSD, these scores were aggregated using a logistic regression model that incorporated demographic factors including age and sex. The logistic regression classifier was trained to achieve 95% specificity, and samples that surpassed the predefined threshold were labeled as cancerous. A weighted ensemble approach was used to determine the most likely cancer type from individual conditional probabilities to predict the TOO. We then successfully identified the top two candidates (top two predictions) and the top predicted cancer type (top accuracy) to assess the performance. Our approach leverages complementary information from multiple cfDNA modalities by building a robust single-feature classifier and integrating it into an ensemble framework.

### TF estimation using ichorCNA

The tumor fraction (TF) was determined using ichorCNA (v0.3.1), a computational tool specifically developed for ultra-low-pass whole-genome sequencing. The TF was estimated on the basis of somatic copy number alterations (SCNAs), which reflect the ctDNA contribution in plasma samples^44,45^. IchorCNA uses a probabilistic model to distinguish tumor-derived SCNAs from the background noise caused by normal cfDNA fragments or technical artifacts. Samples with TFs below 3% were classified as having ‘low TF’, whereas those exceeding 10% were classified as ‘high TF’. To account for potential confounding factors such as clonal hematopoiesis, genomic regions commonly altered in healthy individuals were excluded from the analysis.

### Statistical analysis

Unsupervised clustering was performed using principal component analysis (PCA) uniform manifold approximation and projection (UMAP). Receiver operating characteristic curve analysis was conducted to evaluate model performance. All statistical analyses were performed using R software (version 4.1.0) and Python (version 3.8.5). Statistical significance was set at a FDR of <0.05 for all analyses.

## Results

### Study design and cohort overview

**Fig. 1:**
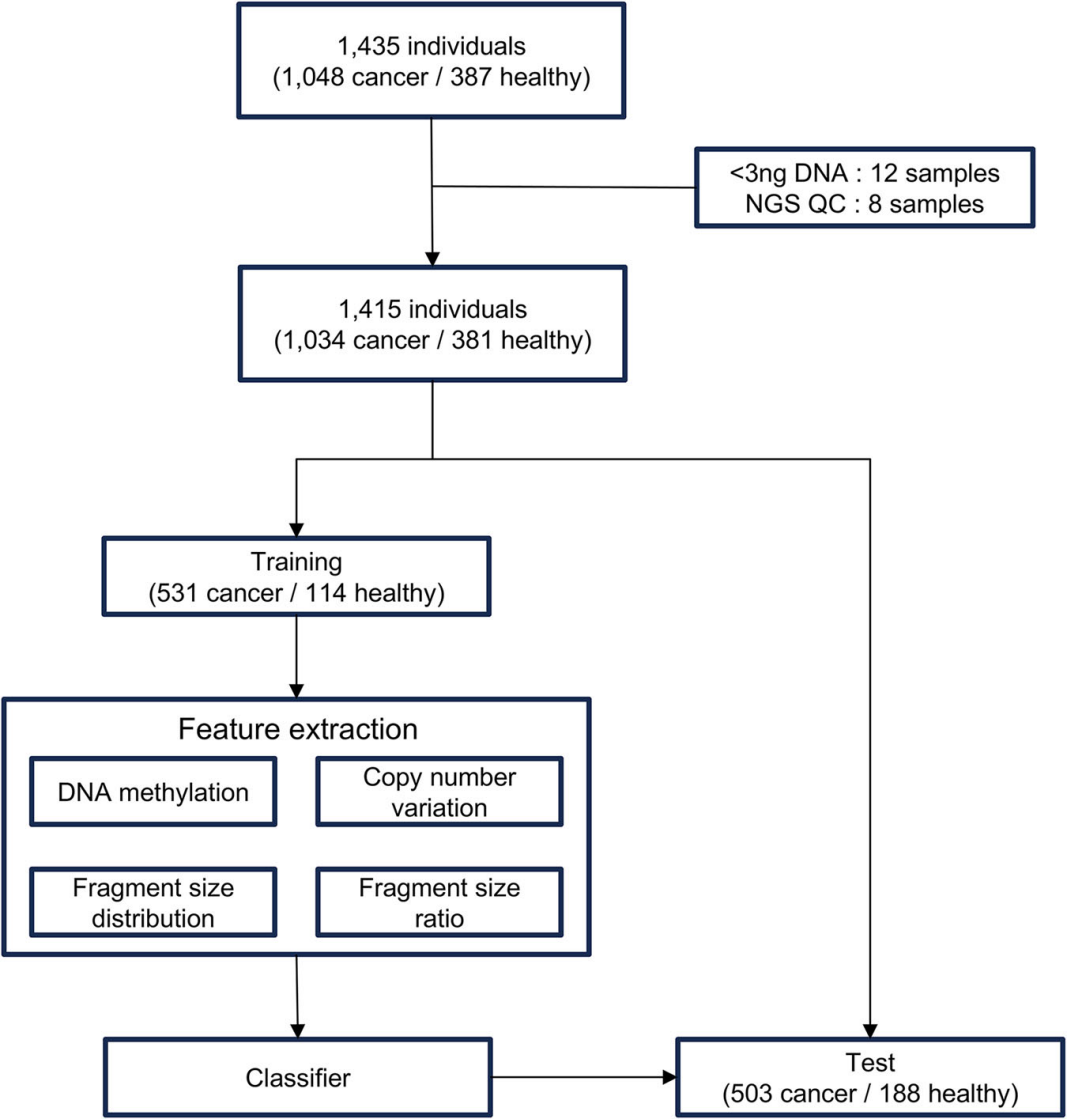
Workflow for MCED model development and validation. The study workflow consisted of four main steps: (1) sample collection and quality control (QC), (2) data partitioning, (3) feature extraction and model development and (4) model evaluation.

This retrospective study used the methodology depicted in Fig. 1 (the study workflow) and incorporated plasma samples from 1048 patients diagnosed with eight prevalent cancer types (colorectal, gastric, liver, pancreatic, lung, breast, ovarian and prostate) as well as whole blood samples from 387 healthy individuals. All participants provided written consent, and the research plan received approval from the institutional review board of the involved institutions (Supplementary Table 1). The final analysis, following quality control filtering, comprised 1415 samples, which included 1034 cancer samples and 381 healthy controls.

Table 1 presents a comprehensive overview of the clinical and demographic characteristics of this cohort. The mean age of the patient cohort was 58.6 years. One crucial step in selecting the healthy control group involved restricting the age range to individuals aged 50 years and older for model training. This was conducted to ensure that the demographics of the target screening population aligned with those of the individuals being screened. The increased risk of false positives with age necessitates the avoidance of age-related methylation drift and other nonmalignant somatic changes. The model training exclusively involved individuals aged 50 years and older who were healthy. In the initial phases of identifying methylation regions and selecting biomarkers, samples from a broader age range were utilized to effectively characterize the noise within the pertinent epigenetic context.

**Table 1:** Characteristics of participants.

Variables, *n*	Training set									
	Healthy <50 years	Healthy ≥50 years	Colon	Gastric	Liver	Pancreas	Lung	Breast	Ovary	Prostate
*N*	79	114	62	55	75	71	120	49	48	51
Age, mean ± s.d. (years)	41.63 ± 6.92	59.19 ± 6.84	61.95 ± 9.41	59.73 ± 12.68	60.47 ± 11.41	63.86 ± 10.35	65.93 ± 11.34	52.98 ± 11.76	54.33 ± 13.82	69.92 ± 7.49
Sex, *n* (%, male)	35 (44.3)	35 (30.7)	39 (62.9)	31 (56.4)	61 (73.5)	42 (59.2)	83 (69.2)	0 (0.0)	0 (0.0)	51 (100)
Stage, *n*										
Stage I			10	23	19	13	52	5	11	1
Stage II			14	16	33	27	19	30	3	34
Stage III			13	14	12	19	6	7	16	10
Stage IV			25	2	11	12	43	7	18	6
	**Test set**									
*N*	79	109	59	45	80	47	131	46	46	49
Age, mean ± s.d. (years)	41.06 ± 7.15	58.64 ± 6.93	61.49 ± 12.00	59.91 ± 12.68	62.01 ± 11.01	63.91 ± 10.35	57.69 ± 11.80	54.24 ± 13.21	54.57 ± 13.20	70.90 ± 7.17
Sex, *n* (%, male)	35 (44.3)	30 (27.5)	35 (59.3)	26 (57.8)	59 (73.8)	26 (55.3)	90 (68.7)	0 (0.0)	0 (0.0)	49 (100)
Stage, *n*										
Stage I			13	22	29	2	36	6	10	0
Stage II			13	13	39	16	4	20	2	34
Stage III			16	7	8	5	5	4	17	10
Stage IV			17	3	4	24	86	16	17	5

The patients with cancer cohort was intentionally designed to emphasize early stage disease (stages I and II) to facilitate a thorough assessment of the assay’s sensitivity in contexts characterized by low ctDNA levels. Among the patients with cancer, 570 (55.1%) were diagnosed with stage I or stage II disease. This indicates that the group is well suited for MCED testing. The cohort demonstrated demographic patterns aligned with epidemiological findings; specifically, breast and ovarian cancers primarily manifested in the early 50s, whereas prostate cancer was noted in the mid-70s. Furthermore, notable proportions of male participants were observed in non-gender-specific cancers, as illustrated by 73% for liver cancer, consistent with recognized prevalence patterns.

Participants were divided into two groups using stratified sampling: a training set and a test set. The ratio of 6:4 indicated a balanced representation of men and women, individuals of varying ages and those at all stages of cancer (I–IV) within both datasets. Supplementary Table 1 provides detailed and precise sample counts for each cancer type and stage. The cohort design facilitates a robust assessment of early stage detection; nonetheless, we acknowledge the absence of stage I pancreatic cancer samples (refer to Table 1 for more details). Despite the insufficient number of samples from the initial seven cancer types, the adequate quantity of early stage samples enabled a comprehensive assessment of the assay’s capability to detect early stage cancer.

### Analysis of unsupervised clustering

Unsupervised clustering was conducted to analyze the inherent structure of cfDNA characteristics and determine their efficacy in differentiating patients with cancer from healthy individuals. This analysis offers essential insights into the contributions of specific features to CSD and guides future model development. Dimensionality reduction techniques, such as UMAP^46^ and PCA^47^, have been used to visualize clustering patterns across four essential features: AMF, CNV, FSR and FSD. The selected features effectively captured tumor-specific signals while minimizing interference from nontumor-derived fragments.

**Fig. 2:**
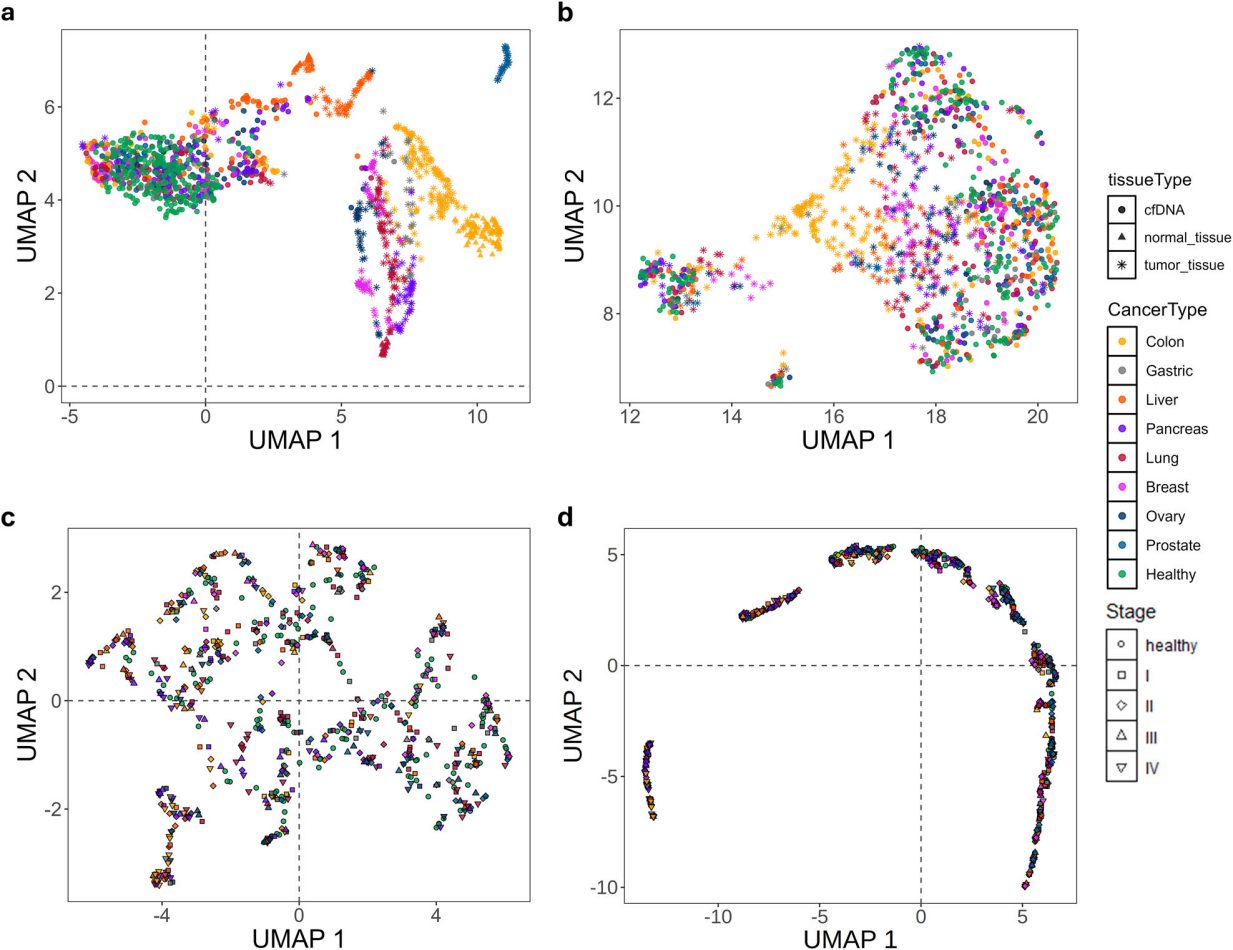
Dimensionality reduction analysis of features in cancer detection. **a** A UMAP visualization of cfDNA and tissue samples based on AMF. Healthy cfDNA samples are shown in green, whereas cfDNA from eight cancer types is color-coded: colorectal (yellow), gastric (gray), liver (orange), pancreatic (purple), lung (red), breast (pink), ovarian (navy) and prostate (blue). Normal tissue samples are represented as triangles and tumor tissue samples as stars. **b**–**d**, The UMAP visualizations of cfDNA samples based on CNV (**b**), FSD (**c**) and FSR features (**d**). The UMAP analyses were conducted using 2.4 million AMF marker regions, 294,000 CNV marker regions, 30 FSD bins and 25,000 FSR regions.

UMAP analysis of AMF demonstrated clear clustering patterns differentiating tumor tissue samples from cfDNA samples. Cancer type-specific clusters were formed in tumor tissues, indicating distinct methylation signatures linked to individual cancers (Fig. 2a). By contrast, cfDNA samples from healthy individuals formed a dense central cluster, indicating consistent methylation profiles among the noncancerous samples. cfDNA samples from patients with cancer demonstrated increased dispersion, with advanced-stage cancers exhibiting more distinct subgroups than early stage cancers. This trend indicates that methylation changes in ctDNA intensify with cancer progression, leading to more a distinct differentiation from healthy cfDNA.

Comparable clustering patterns were identified using the CNV-based UMAP analysis. Samples from patients with late-stage cancer exhibited distinct clustering, differentiating them from healthy individuals and patients with early stage cancer, thereby underscoring the progressive accumulation of genomic alterations associated with increased tumor burden (Fig. 2b). Fragmentomic characteristics exhibited complementary clustering patterns. FSD-based clustering revealed a partial overlap between healthy individuals and patients with cancer; however, samples from stages II–IV tended to cluster in specific regions, particularly within the lower-left quadrant of the plot (Fig. 2c). FSR analysis indicated a moderate distinction between healthy and cancer cfDNA samples, with late-stage cancers grouped on the left-hand side of the projection (Fig. 2d).

The results of PCA supported the findings from UMAP, highlighting the increasing divergence of cfDNA profiles as the tumor burden progresses. Late-stage cancers exhibited distinct clustering, whereas early stage cancers showed some overlap with the healthy cfDNA samples. The results indicated that ctDNA levels increased as tumor size increased, leading to greater DNA shedding into the bloodstream (Supplementary Fig. 5).

The biological implications of our findings are important. The clustering observed in advanced-stage cancers probably indicates increased ctDNA shedding resulting from a higher tumor burden, whereas the partial overlap between early stage cancers and healthy individuals highlights the challenges in detecting subtle tumor-derived signals in low-ctDNA contexts. These observations underscore the necessity for multimodal strategies that incorporate various cfDNA characteristics to improve the sensitivity of early stage cancers.

Unsupervised clustering analysis revealed that methylation-based (AMF), genomic alteration (CNV) and fragmentomic (FSR and FSD) features offer complementary insights into tumor biology. These findings highlight the potential for incorporation of MCEDs into a multimodal framework.

### Differentially methylation regions among tumor tissue, cfDNA and healthy cfDNA

Genome-wide methylation patterns were analyzed in tumor tissues, cfDNA from patients with cancer and healthy cfDNA to identify cancer-specific methylation markers. DMRs were identified through three sequential comparisons: (1) tumor tissue versus adjacent normal tissue, (2) tumor tissue versus healthy cfDNA and (3) cfDNA from patients with cancer versus healthy cfDNA. Statistical significance was assessed using *t*-tests, applying a FDR threshold of less than 0.05. The selection of DMRs was optimized by assessing directional concordance, which necessitates uniform methylation alterations between the tissue and cfDNA. DMRs have been identified in colorectal, liver and lung cancers by comparing cancerous and adjacent normal tissues. In cancers without matched normal tissue, such as pancreatic and ovarian cancers, DMRs were identified by comparing cfDNA from patients with cancer with that from healthy individuals and subsequently applying concordance filtering using tissue-derived markers.

**Fig. 3:**
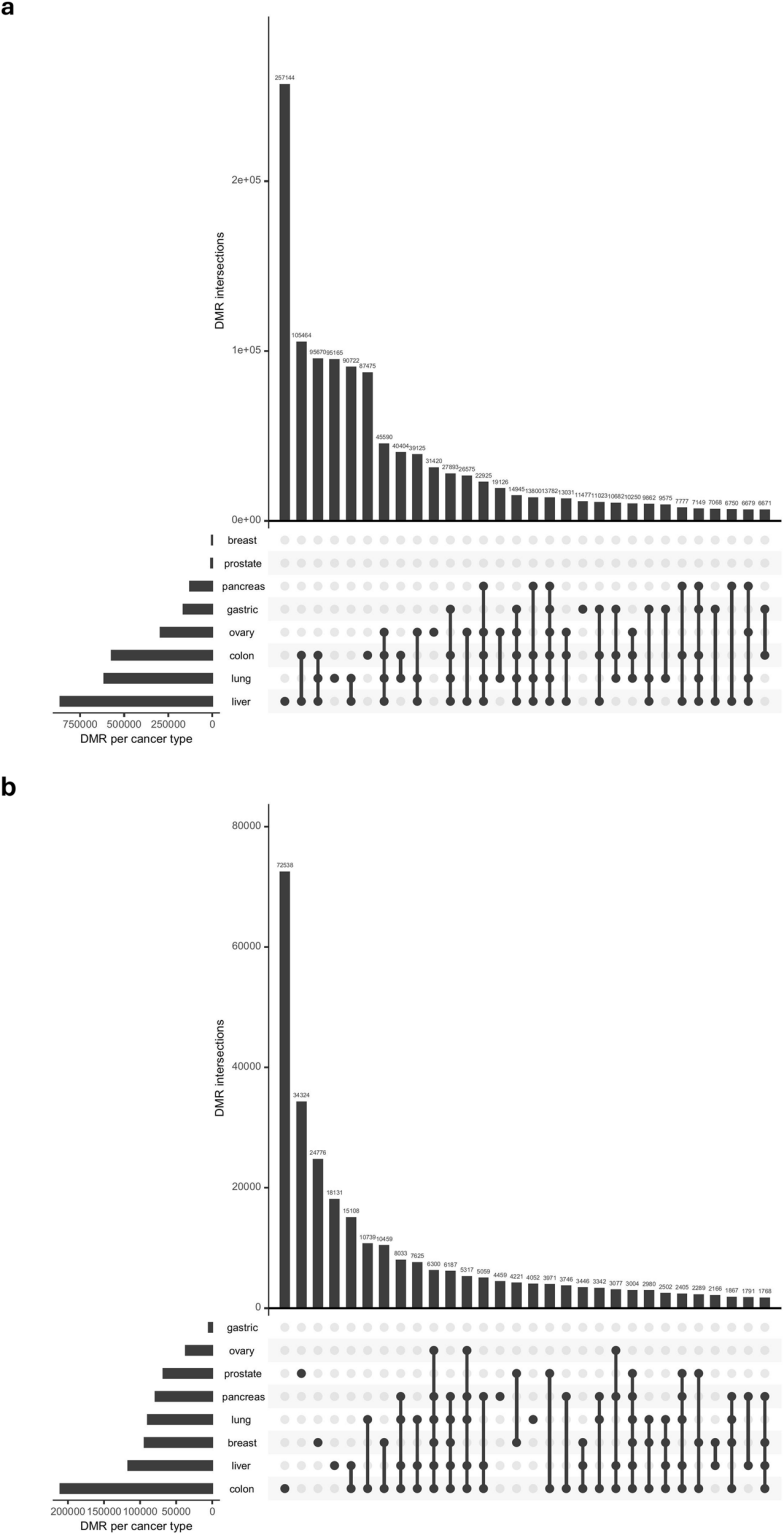
Upset plot for DMR selection. **a**, **b** The UpSet plots of markers by cancer type for hypo- (**a**) and hypermethylated markers (**b**) selected by DMR. For each cancer type, a graph showing the intersection of markers selected by comparing cancer tissue and normal cfDNA and cfDNA from patients with cancer and normal cfDNA. Top: the bar plot shows the number of intersection markers. Bottom: the dot plot shows the cancer type belonging to that bar. Bottom left: the bar plot shows the number of DMRs for that cancer type.

An analysis of UpSet plots demonstrated clear patterns of hypo- and hypermethylated DMRs across various cancer types (Fig. 3 and Supplementary Fig. 6). Liver cancer displayed the greatest quantity of unique hypo-DMRs, totaling 1,200 regions, which probably indicates its distinct epigenetic landscape marked by the activation of the Wnt–β-catenin pathway. By contrast, gastric cancer exhibited no hyper-DMRs, indicating a distinct methylation regulation. Approximately 18% of hypo-DMRs and 12% of hyper-DMRs are common across three or more cancer types, suggesting the presence of pancancer epigenetic signatures. Shared hyper-DMRs in tumor suppressor gene promoters, such as CDKN2A, have been identified in colorectal, lung and breast cancer.

These findings underscore the presence of both cancer-specific and pancancer methylation signatures and establish a solid basis for multimodal MCED frameworks. The combination of tissue-guided and cfDNA-specific DMRs overcomes the limitations associated with single-feature methodologies, as evidenced by recent studies.

### Performance of single-feature classifiers in CSD and TOO prediction

Machine learning models were developed for each cfDNA feature to assess their diagnostic utility by classifying samples as healthy or cancer positive. These single-feature classifiers were evaluated on the basis of sensitivity, maintaining a specificity threshold of 95%.

Among the single-feature classifiers, AMF exhibited the highest sensitivity, reaching an overall sensitivity of 85.3% (95% confidence intervals (CI), 81.9–88.3; Supplementary Fig. 7). AMF identifies global methylation patterns in specific CpG regions, demonstrating high sensitivity for early stage cancers characterized by low ctDNA levels. AMF demonstrated sensitivities of 98.3% and 93.5% for colorectal and breast cancers, respectively. The capacity to identify subtle tumor-derived methylation signals has been notably demonstrated in difficult cases, including ovarian and prostate cancers, which typically show low ctDNA abundance.

CNV analysis plays a crucial role in cancer detection by identifying the genomic alterations associated with tumor progression. At the single-feature level, CNV demonstrated an overall sensitivity of 60.6% (95% CI, 56.2–64.9; Supplementary Fig. 7), exhibiting particularly robust performance for colorectal cancer (93.2%) and breast cancer (91.3%). The exclusion of genomic regions frequently modified in healthy individuals enhances the classification accuracy by minimizing false positives. Late-stage cancers demonstrate enhanced CNV signals attributable to elevated tumor burden, thereby increasing the observable copy number alterations (Materials and methods).

The fragmentomic characteristics offer an additional understanding of cfDNA biology. The FSR, which measures the ratio of short fragments (greater than 80 bp and less than 150 bp) to long fragments (greater than 150 bp and less than 220 bp), demonstrated an overall sensitivity of 67.8% (95% CI, 63.5–71.9; Supplementary Fig. 7). This feature demonstrated notable efficacy in colorectal cancer (86.4%) and breast cancer (95.7%), as ctDNA fragmentation patterns exhibited notable differences compared with healthy individuals. FSD, which examines the distribution of cfDNA fragment sizes within specified bins, attained an overall sensitivity of 70.2% (95% CI, 66.0–74.1; Supplementary Fig. 7). This method demonstrated notable efficacy in identifying cancers characterized by specific fragmentation profiles, including liver cancer (81.3%) and breast cancer (80.4%).

The efficacy of single-feature classifiers differed according to cancer type and stage. Digestive organ cancers, including colorectal, gastric, liver and pancreatic cancers, have lower sensitivity in stage III than in stages I and II. This observation aligns with previous studies, indicating that lymph node metastasis influences ctDNA-shedding dynamics at this stage. Regarding the TOO prediction accuracy, CNV attained the highest top 1 accuracy of 71.9% (Supplementary Fig. 8b), followed by AMF at 58.2% (Supplementary Fig. 8a), FSR at 46.7% (Supplementary Fig. 8c) and FSD at 50.3% (Supplementary Fig. 8d). The FSD exhibited robust performance in lung cancer detection, attaining a recall rate of 73.8%.

These findings highlight the necessity of incorporating various cfDNA characteristics to improve the diagnostic precision and reliability across different cancer types and stages. AMF has emerged as the most important feature owing to its capacity to detect subtle tumor-derived methylation signals. However, the complementary roles of CNV, FSR and FSD underscore their importance within a multimodal framework of MCED.

### CSD model utilizing ensemble methods

**Fig. 4:**
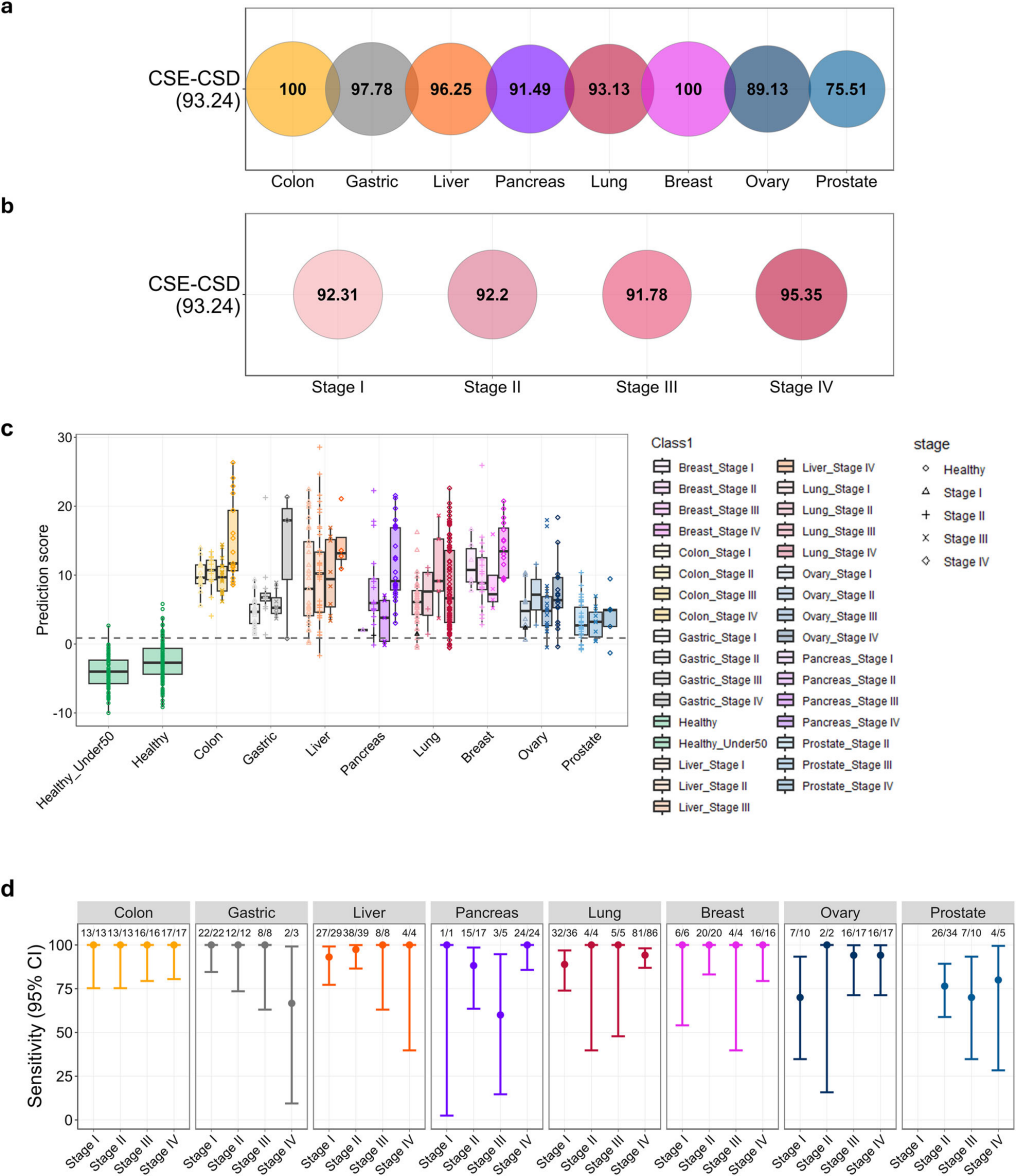
CSE–CSD performance for the test dataset. **a** The bubble plot illustrates the performance of the CSE–CSD model on the test dataset. The sensitivity for each cancer type is represented by the size and numerical value of the bubbles, whereas overall sensitivity is indicated on the *y* axis label (the unit is percentage). **b** The bubble plot showing the CSE–CSD rates for different cancer stages. The size and color intensity of each bubble correspond to the detection rate, with larger and darker bubbles indicating higher detection rates. **c** A box plot showing the distribution of performance scores for healthy individuals and patients with cancer. To indicate cancer stages, circle dots represent healthy samples, whereas stage I, stage II, stage III and stage IV cancers are represented by triangles, crosses, X marks and diamonds, respectively. The dashed gray line indicates the threshold determined during training. **d** The dot plot displays sensitivity for each cancer type across stages with 95% confidence intervals. The total number of samples and the number of detected cancer samples for each stage are annotated at the top of the plot.

A CSE–CSD model was created by incorporating various cfDNA features, including AMF, CNV, FSR and FSD, within an ensemble machine learning framework. This multimodal approach aims to utilize the complementary strengths of these features to improve the sensitivity and specificity of MCED. The ensemble model exhibited strong performance across the eight cancer types, attaining an overall sensitivity of 93.2% with a fixed specificity of 95% (Fig. 4a). Sensitivity was consistently high across all cancer stages, with stage I cancers demonstrating a sensitivity of 92.3%, underscoring the model’s capability to identify early stage tumors, which generally exhibit low ctDNA levels. The sensitivity for stage II cancers was 92.2%, whereas that for stage III cancers exhibited a minor reduction to 91.8%, consistent with the trends observed in single-feature models. The sensitivity increased to 95.4% for stage IV cancers, which was attributed to the elevated tumor burden and enhanced ctDNA-shedding rates (Fig. 4b).

An analysis of cancer type-specific stage performance revealed that stage III sensitivity in gastrointestinal cancers (colorectal, gastric, liver and pancreatic) is slightly lower than that of stages I and II (Fig. 4b). This observation is consistent with findings that indicate temporary alterations in ctDNA-shedding dynamics during lymph node metastasis. The ensemble model effectively compensated for this minor decline. The AMF feature was crucial for early stage detection as it identified initial epigenetic signals. In stage III, the CNV and fragmentomics features compensated by utilizing signals from accumulated genomic instability, thereby maintaining a consistent diagnostic accuracy across all stages of progression.

The sensitivity specific to cancer varied among the eight analyzed types. Colorectal and breast cancer demonstrated the highest sensitivity (100.0%), followed by gastric cancer (97.8%). The model demonstrated sensitivities of 91.5%, 89.1% and 75.5% for pancreatic, ovarian and prostate cancers, respectively, which currently lack established screening guidelines. These findings underscore the clinical applicability of the CSD model in identifying difficult cancer types.

The distribution of the CSE–CSD scores across cancer stages exhibited notable trends. In Fig. 4c, healthy individuals consistently demonstrated low scores, establishing a closely grouped baseline, whereas patients with cancer exhibited progressively higher scores as the stages advanced. Stages I and II cancers exhibited notable differentiation from healthy individuals, highlighting the capacity of the model to identify tumors with low ctDNA levels. A subset of stage III cancers overlapped with earlier stages or healthy samples, indicating variability in ctDNA-shedding dynamics.

The 95% confidence interval for sensitivity across cancer types and stages indicated that colorectal cancer exhibited consistently high sensitivity, irrespective of stage. The sensitivity generally increased with stage for all cancer types, whereas lung cancer demonstrated comparable sensitivity and confidence intervals between stages I and IV. The confidence interval for pancreatic cancer was notably wide owing to the presence of only one independent test sample for stage I (Fig. 4d).

The ensemble framework successfully mitigated the limitations identified in the single-feature models by utilizing the strengths of AMF, CNV, FSR and FSD features. AMF captures tumor-specific methylation signals while minimizing interference from nontumor cfDNA fragments, thereby improving the sensitivity of early stage cancers, including ovarian and prostate cancer. CNV analysis enhances the classification accuracy for colorectal and breast cancers through the identification of tumor-specific genomic alterations. By contrast, FSR and FSD contributed additional discriminatory power by utilizing variations in cfDNA fragmentation patterns between tumor-derived and normal cfDNA fragments.

These findings highlight the significance of incorporating various cfDNA characteristics to improve diagnostic accuracy and reliability across different cancer types and stages. This study integrated methylation profiling, genomic alterations and fragmentomic characteristics within an ensemble framework, resulting in notable improvements in the sensitivity, specificity and accuracy of TOO prediction of MCED.

Our integrated multimodal ensemble model (CSE–CSD) utilizes a logistic regression framework that amalgamates the prediction probabilities from the four constituent cfDNA features: AMF, CNV, FSR and FSD. This ensemble architecture was created to get around the problems with single-feature assays and make the most of the information that each modality adds.

We looked at the coefficients of the underlying logistic regression model to see how much each feature added to the final CSD score. These coefficients show how important and heavy each input is. The AMF feature had the highest positive coefficient, which meant that it was the most important factor in the final CSD score. This finding indicates that AMF has the best discriminatory ability to identify epigenetic alterations associated with tumorigenesis. The CNV, FSR and FSD features also had positive coefficients, which showed that they were very important in adding to the AMF signal to improve the overall detection accuracy and reliability.

Supplementary Table 5 presents the exact coefficient values (weights) that were given to the normalized probabilities of each feature model. This quantitative analysis elucidates the ‘black box’ aspect of the ensemble operation and offers a mechanistic comprehension of how the amalgamation of features attains synergy, especially in the demanding realm of early stage cancer detection marked by low ctDNA abundance.

### Ensemble TOO prediction model performance

TOO prediction is essential for MCED, enabling clinicians to determine the primary site of cancer in samples identified as cancer positive. This study presents the development of a CSE–TOO prediction model through a multimodal framework that incorporates various cfDNA features. The model exhibited strong performance across eight cancer types—colorectal, gastric, liver, pancreatic, lung, breast, ovarian and prostate—by utilizing the complementary strengths of these features.

**Fig. 5:**
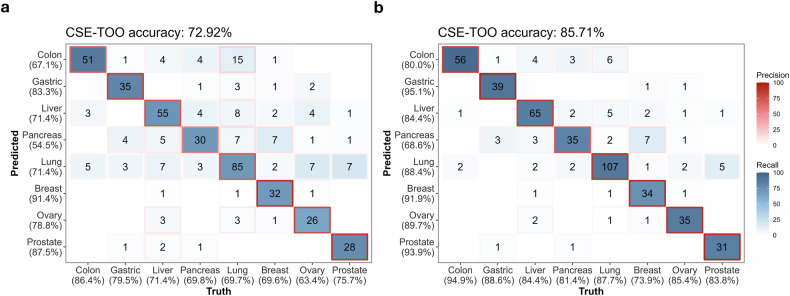
CSE–TOO prediction performance for the test dataset. **a**, **b** Top 1 (**a**) and top 2 (**b**) confusion matrices showing TOO prediction performance. **a** shows the top 1 prediction, whereas **b** shows the top 2 predictions. The confusion matrices depict relationships between the predicted class (*y* axis) and actual cancer type (*x* axis) for eight cancer types. The blue gradient shading within cells indicates recall, whereas the red gradient borders represent precision. The overall TOO prediction accuracy (%) is shown at the top of each matrix.

The CSE–TOO prediction model attained a top 1 accuracy of 72.9%, with the highest probability of the cancer type being deemed correct. Incorporating the second-highest probability into the evaluation (top 2 accuracy) resulted in a remarkablet performance enhancement of the model, achieving an accuracy of 85.7%. These findings underscore the importance of incorporating secondary predictions when cfDNA signals overlap across different cancer types. The performance across different cancer types exhibited notable differences, with colon cancer attaining the highest top 1 recall of 86.4%, which was attributed to the robust AMF signal that reflects a tumor-specific methylation pattern (Fig. 5a and Supplementary Fig. 8a). It was the only entry in top 2 to attain a recall value exceeding 90%, achieving 94.9% (Fig. 5b). Gastric cancer (79.5%) and prostate cancer (75.7%) showed notable accuracies. Conversely, breast cancer demonstrated the highest precision at 91.4%, suggesting that the samples identified as breast cancer were most likely accurate. Prostate cancer (87.5%), followed by gastric cancer (83.3%), displayed a sequence similar to the recall. In the top 2 rankings, gastric cancer (95.1%), prostate cancer (93.9%) and breast cancer (91.9%) achieved high positions (>90%), highlighting a important finding, as these cancers are typically challenging to detect using cfDNA.

To resolve ambiguous classifications with closely matched probabilities for multiple cancer types, thresholds were implemented on the basis of the difference between the highest and second-highest probabilities. When a threshold of 0.07 was applied, the top 1 prediction accuracy increased to 80.3%, and the top 2 accuracy increased to 89.24% (Supplementary Fig. 9b–e). Approximately 10–20% of the samples were categorized as ‘in between’ under these thresholds, indicating uncertainty in the CSE–TOO predictions for those instances (Supplementary Fig. 9a).

Each cfDNA feature made a distinct contribution to the prediction performance of the CSE–TOO. AMF have identified global methylation patterns in specific CpG regions, demonstrating high sensitivity for early stage cancers. CNV plays a crucial role in identifying genomic alterations pertinent to tumor biology, thereby enhancing the classification accuracy for cancers, including colorectal and breast cancers. FSR and FSD offer complementary insights by utilizing variations in cfDNA fragmentation profiles between tumor-derived and normal cfDNA fragments.

These findings highlight the clinical relevance of the CSE–TOO prediction model in identifying specific cancer types, thus minimizing the need for extensive diagnostic evaluations. Lung cancer prediction with a high FSD probability may direct clinicians to utilize low-dose CT scans, whereas colorectal cancer prediction exhibiting strong AMF signals could necessitate colonoscopy confirmation.

The CSE–TOO prediction model effectively used multimodal cfDNA features, resulting in a high accuracy in predicting CSE–TOO across various cancer types. The strong performance underscores its potential utility in directing follow-up diagnostic procedures within MCED frameworks while also addressing crucial deficiencies in existing screening practices.

### Feature contribution analysis and comparison of CSD scores

Feature contributions were analyzed to assess the diagnostic value of individual cfDNA features (AMF, CNV, FSR and FSD) and their incorporation into the ensemble framework for predicting CSE–CSD and CSE–TOO. Each feature uniquely contributed to the overall performance of the model, offering complementary insights into tumor biology and improving diagnostic accuracy across various cancer types and stages.

AMF has become an essential tool for analyzing global methylation patterns in specific CpG regions. It exhibits notable sensitivity for early stage cancers by quantifying the average methylation levels within these regions, where ctDNA is present in low abundance. AMF demonstrated sensitivities of 98.3% and 93.5% for colorectal and breast cancers, respectively. The capacity to identify subtle tumor-derived methylation signals has been notably demonstrated in difficult cases, including ovarian and prostate cancers, which typically show low rates of ctDNA shedding.

CNV analysis enhances classification accuracy by identifying genomic alterations linked to tumor progression. Excluding genomic regions that are frequently altered in healthy individuals and concentrating on gene-based regions improves the sensitivity of CNV analysis for cancers characterized by unique genomic signatures. CNV demonstrated sensitivities of 93.2% and 91.3% for colorectal and breast cancers, respectively. Late-stage cancers demonstrate enhanced CNV signals because of elevated tumor burden, thereby increasing the visibility of copy number alterations.

The fragmentomic characteristics offer an additional understanding of cfDNA biology. FSR utilizes the differences in fragmentation patterns between tumor-derived and normal cfDNA fragments. The FSR demonstrated an overall sensitivity of 67.8%, with notable performance in colorectal cancer (86.4%) and breast cancer (95.7%). The FSD evaluates the distribution of cfDNA fragment sizes across specified bins, resulting in an overall sensitivity of 70.2%. FSD demonstrated notable efficacy in identifying cancers characterized by specific fragmentation profiles, including liver cancer (81.3%) and breast cancer (80.4%).

**Fig. 6:**
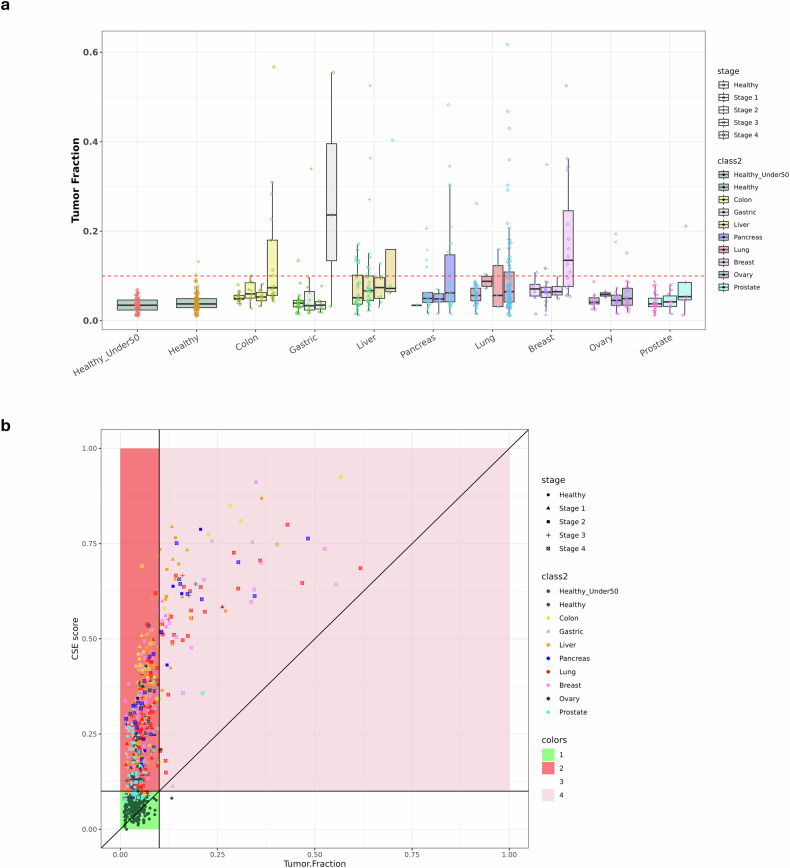
TF analysis. **a** The TF distribution by cancer types and stage and healthy individuals. Each box indicates the stage of the relevant cancer, the *y* axis shows the TF determined by ichorCNA and the *x* axis denotes cancer. The cutoff point (0.1) for identifying cancer in ichorCNA is represented by the reference line. **b** A scatter plot illustrating the correlation between the CSE–CSD score and TF. The horizontal and vertical lines, as shown in **a**, are represented by the reference line. Red indicates high areas only in the CSE–CSD score, pink indicates high TF values in both values and green indicates low TF values in both levels.

To determine whether cancer signals could be detected at low TFs, the correlation between the amount of tumor-derived DNA and multimodal CSE–CSD scores was assessed by predicting the TF using ichorCNA, a computational tool for analyzing ultra-low-pass whole-genome sequencing data. Figure 6a illustrates the distribution of TF levels across all samples, categorized by cancer type and stage. The tumor frequency of stage I and II tumors across all cancer types was comparable to that of healthy individuals; however, the tumor frequency in several stage III and IV samples exhibited a substantial disparity. The study revealed a substantial positive correlation between the tumor ratio and CSE–CSD scores (Pearson’s *r* = 0.70, *P* < 0.001; Fig. 6b). Advanced-stage (stages III and IV) cancers showed higher TFs (median of 5.9%) and CSD scores (median of 30.6%) than early stage cancers (median TF, 5.1%; median, 26.1%). In stage I cancers, which are characterized by low TFs (<5% in 52.1% of cases), the CSD model demonstrated a sensitivity of 85.1%, indicating its effectiveness in identifying subtle tumor-derived signals that may be overlooked by SCNA-based methods.

In discordant cases, elevated CSE–CSD scores (>0.3) were noted despite low TF (<3%), specifically in breast (*n* = 2) and ovarian (*n* = 1) cancers. These findings indicate that methylation and fragmentomic characteristics offset the scarcity of CNV in these cancers. By contrast, samples exhibiting high TF (>10%) and low CSE–CSD scores (<0.5) were associated with confounding factors including clonal hematopoiesis or inflammatory conditions, underscoring the specificity of the multimodal approach.

These findings highlight the necessity of incorporating various cfDNA characteristics to improve diagnostic precision across different cancer types and stages, while also addressing important deficiencies in existing screening methodologies.

## Discussion

The findings of this study underscore the potential of a multimodal cfDNA analysis framework for MCED. The ensemble model demonstrated high sensitivity across various cancer types and stages by integrating methylation profiling (AMF), genomic alterations (CNV) and fragmentomic features (FSR and FSD), while also ensuring robust specificity. These findings highlight the significance of utilizing complementary cfDNA characteristics to address the limitations identified in single-feature methodologies.

The ensemble model exhibited a sensitivity of 93.2% with a specificity of 95%, surpassing current cfDNA-based techniques, including GRAIL’s Galleri test, which has a sensitivity of approximately 51.5%, and DELFI’s fragmentomics method, which has a sensitivity of approximately 60%. The model demonstrated high efficacy in identifying early stage cancers, achieving a sensitivity of 92.3% at stage I, despite the generally low abundance of ctDNA. This performance is due to the ability of AMF to detect subtle methylation alterations and FSD’s sensitivity of FSD to fragmentation patterns linked to apoptosis-derived cfDNA fragments. Colorectal cancer exhibited the highest sensitivity (98.3%), indicating unique methylation signatures that are easily identifiable through AMF.

The underlying biological mechanism for this performance relies on the synergistic and orthogonal power of the features: AMF is optimized to capture subtle, high-signal-to-noise ratio epigenetic alterations that arise early in oncogenesis, making it crucial for early stage diagnosis. Furthermore, this strategic compensatory system sustains consistent performance and stage-specific resilience (addressing the tendency for lower stage III sensitivity in GI cancers) by having CNV and fragmentomics facilitate detection in later stages (such as stage III), where enhanced signals result from accumulated genomic instability. This integrated approach is key to maintaining stable diagnostic accuracy across all stages.

Although the TOO prediction achieved a top 1 accuracy of 72.9% and a top 2 accuracy of 85.7%, analysis of the remaining samples (14.3%) that did not meet top 2 classification suggests caution in clinical reporting. These ‘in-between’ samples were mainly found in (1) early stage cancers with very low TF or (2) similar tissue types that had a lot of overlap in their epigenetic profiles (for example, gastric and colorectal cancer). To resolve this ambiguity and enhance TOO resolution, future initiatives will focus on increasing the resolution of methylation markers (for example, using single CpG-level data) and strengthening border categorization by broadening the cohort to include rare or challenging tumor types.

Despite these encouraging findings, several challenges remain to be overcome. The sensitivity for cancers characterized by low ctDNA abundance, such as ovarian and prostate cancers, was relatively low, at approximately 89.1% and 75.5%, respectively. This limitation underscores the necessity for the additional optimization of feature selection to improve signal detection in low ctDNA contexts. Stage-specific performance variability was noted, particularly in stage III cancers, where lymph node metastasis may influence ctDNA-shedding dynamics. The cohort size for specific cancer types, such as pancreatic cancer, was relatively small, potentially limiting generalizability to wider populations.

Foremost among these limitations is the inherent risk of optimism bias stemming from the training and testing conducted within a single cohort. We explicitly recognize that the generalizability and ultimate real-world clinical utility of our model mandates rigorous external, independent validation using a newly collected, prospective cohort. To directly address this critical necessity, we are actively conducting a large-scale prospective clinical validation study supported by the Korean Advanced Research Projects Agency. This ongoing trial is specifically designed to rigorously test the assay’s performance and robustness in a real-world screening setting, thereby resolving the potential for optimism bias. The details regarding the funding for this pivotal clinical investigation are provided in the Funding and Acknowledgement sections of this manuscript. We anticipate disseminating the comprehensive findings of this prospective trial in subsequent publications.

Future studies should emphasize prospective validation in larger cohorts to establish generalizability across various populations and clinical contexts. Furthermore, technical advancements, including feature refinement through the integration of novel methylation markers or enhanced fragmentation metrics, may effectively improve the diagnostic performance. Incorporating this into clinical workflows necessitates real-world testing to assess the cost-effectiveness, scalability and compatibility with current diagnostic protocols.

This study demonstrated remarkable advancements in MCED via multimodal cfDNA analysis. This approach has the potential to transform cancer screening practices and enhance early detection outcomes by addressing key limitations and pursuing targeted improvements in diverse patient populations.

## Supplementary information

Supplementary Information

## Data Availability

The raw data for this study were generated by IMBdx Inc. Data supporting the findings of this study are available from the corresponding author upon reasonable request. Data access may be subject to institutional and ethical regulations.
