# Supplementary material for: Enhanced multicancer screening assay through whole-genome methylation sequencing-based multimodal cell-free DNA analysis
Source: Exp Mol Med. 2026 Apr 21;58(4):1311–24. doi: 10.1038/s12276-026-01674-7 (PMC13144671; doi:10.1038/s12276-026-01674-7)
Supplement: Supplementary file 1 — Supplementary Information [file 12276_2026_1674_MOESM1_ESM.pdf]

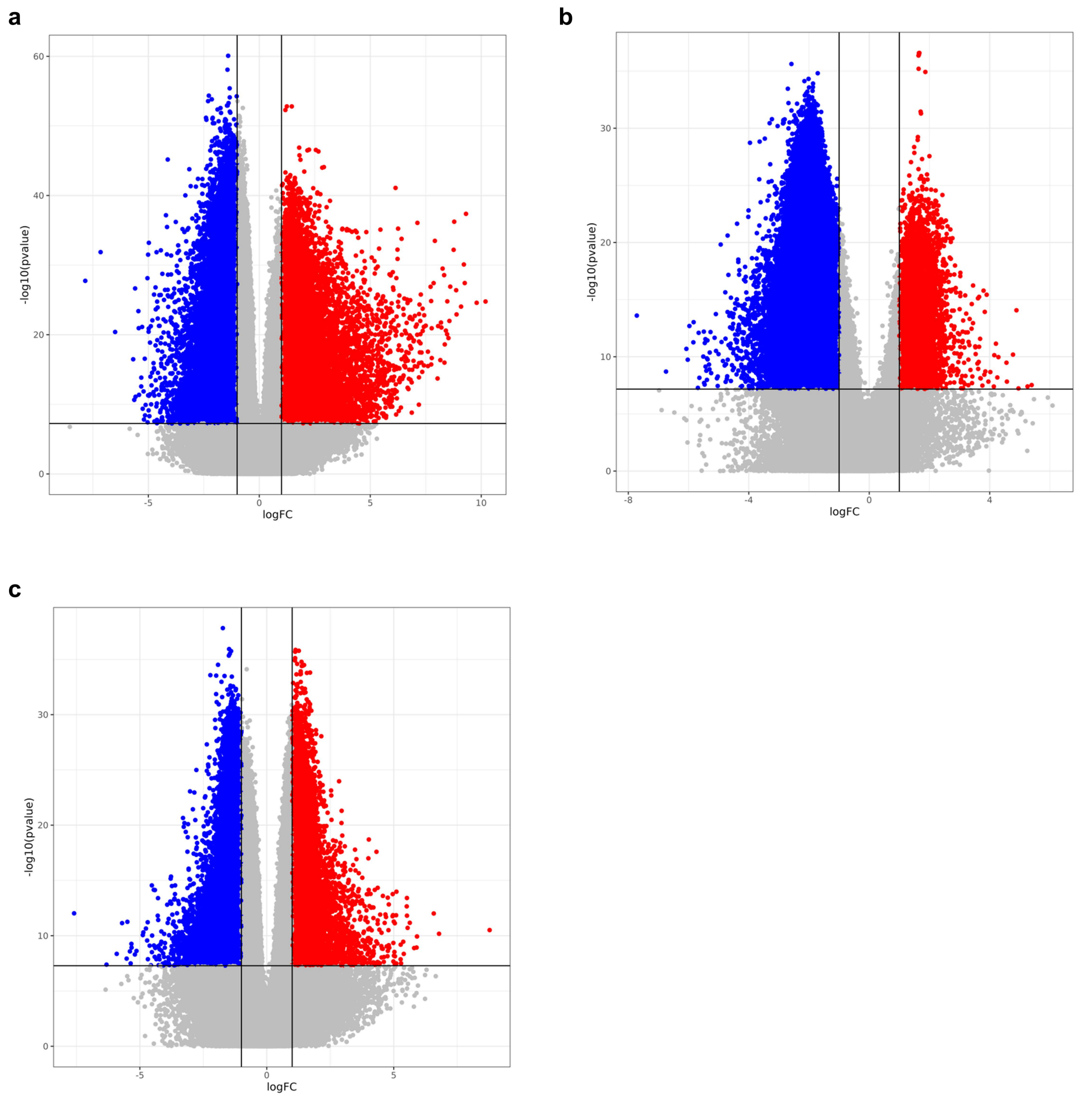

Supplementary Fig. 1 Volcano plot indicating differentially methylated markers between tumor tissue and normal tissue. For each (a) colon, (b) liver, (c) lung cancer type, a differential methylation analysis was conducted comparing the associated tumor tissue samples with the normal tissue. Each volcano plot demonstrates the –log10 p-value against the log2 fold changes. Dot color means the hyper- (red) and hypo-methylation (blue) markers, respectively.

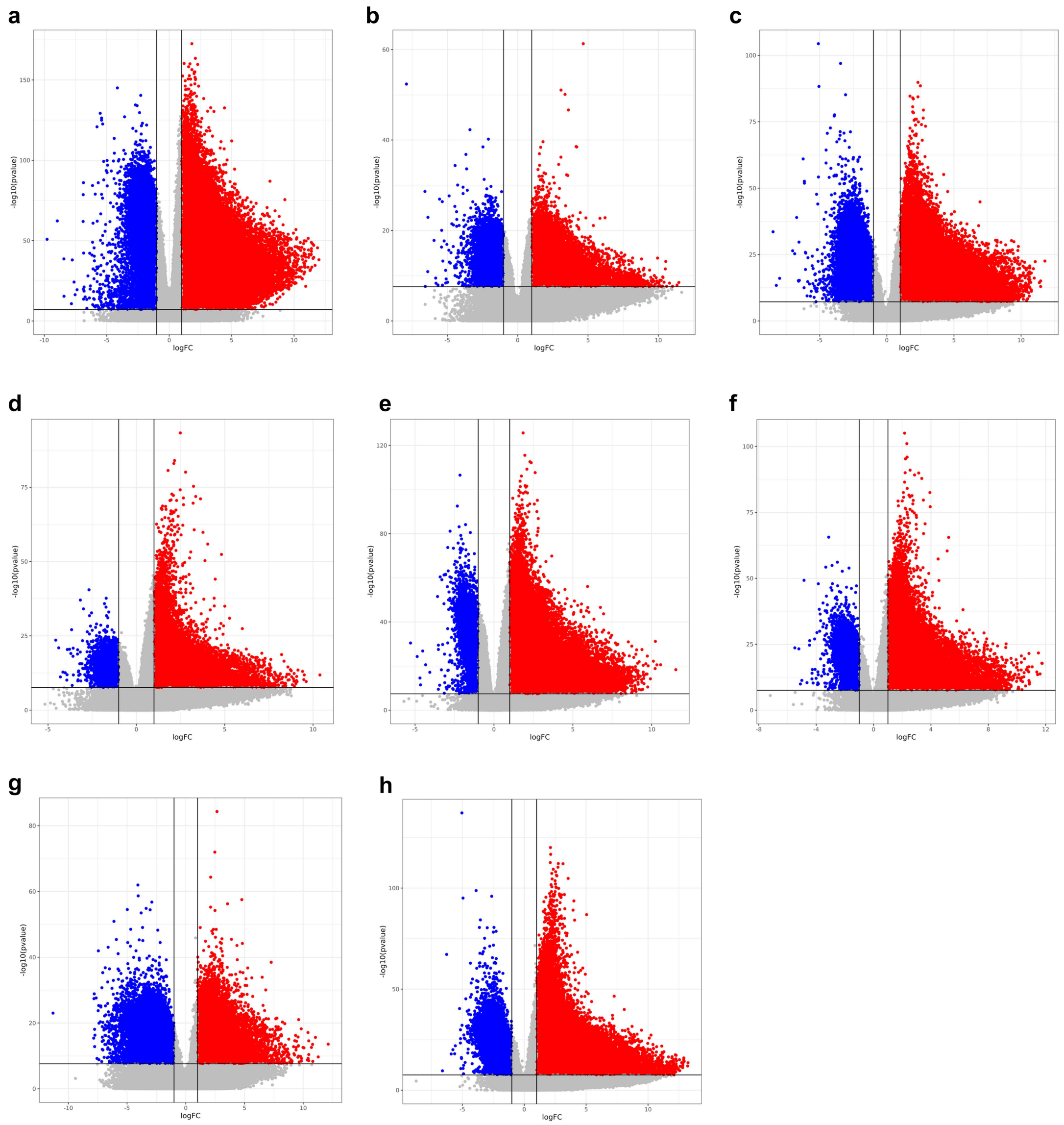

Supplementary Fig. 2 Volcano plot indicating differentially methylated markers between tumor tissue and healthy training cfDNA. For each (a) colorectal, (b) gastric, (c) liver, (d) pancreatic, (e) lung, (f) breast, (g) ovarian, and (h) prostate cancer type, a differential methylation analysis was conducted comparing the associated tumor tissue samples with the normal tissue. Each volcano plot demonstrates the –log10 p-value against the t-test statistics. Dot color means the hyper- (red) and hypo-methylation (blue) markers, respectively.

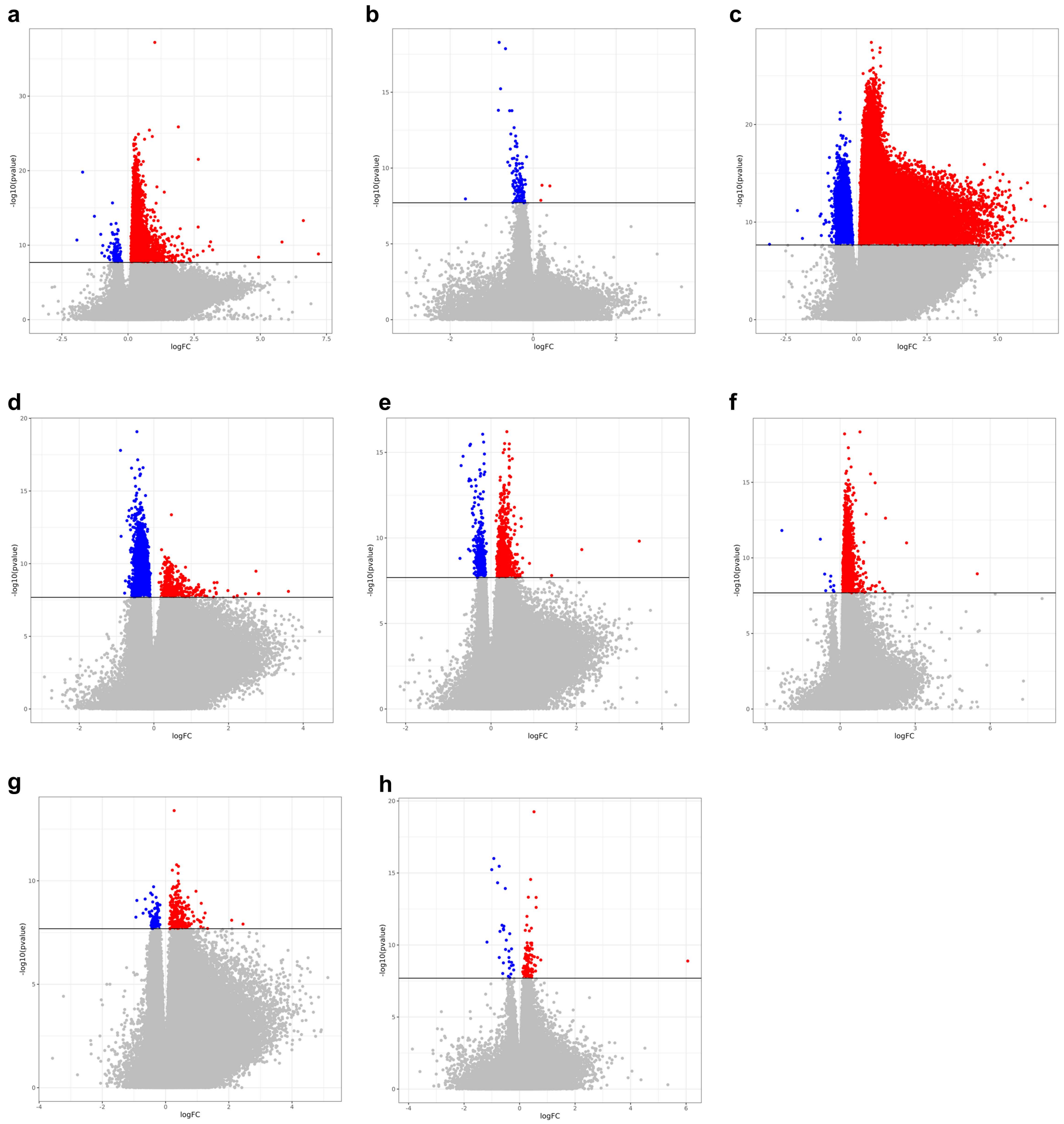

Supplementary Fig. 3 Volcano plot indicating differentially methylated markers between cancer cfDNA and healthy training cfDNA. For each (a) colorectal, (b) gastric, (c) liver, (d) pancreatic, (e) lung, (f) breast, (g) ovarian, and (h) prostate cancer type, a differential methylation analysis was conducted comparing the associated tumor tissue samples with the normal tissue. Each volcano plot demonstrates the –log10 p-value against the t-test statistics. Dot color means the hyper- (red) and hypo-methylation (blue) markers, respectively.

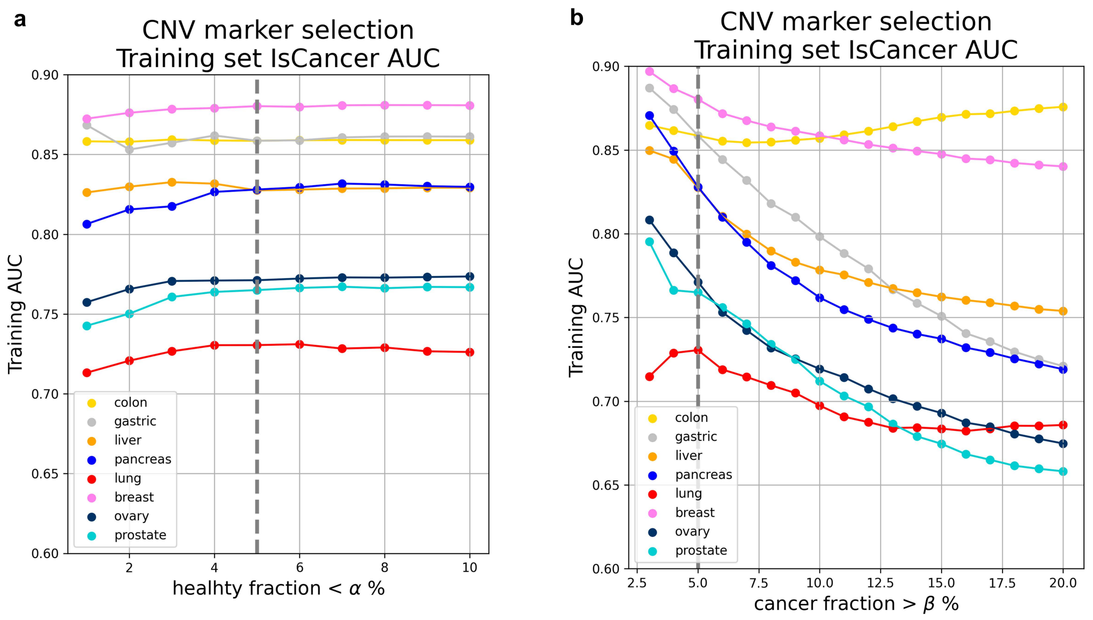

Supplementary Fig. 4 Training set AUC performance by CNV marker selection criteria across cancer types. (a) Cancer fraction > 5% and healthy fraction < α%. (b) Healthy fraction < 5% and cancer fraction > β%

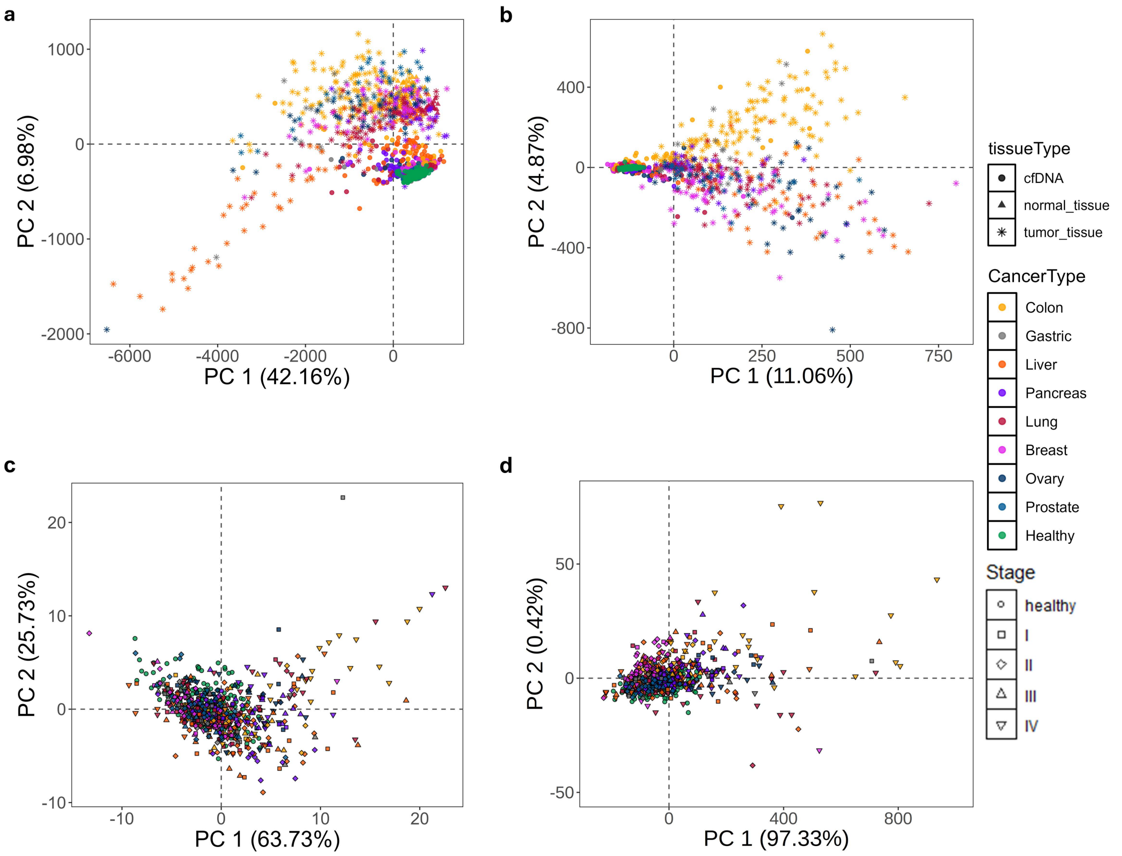

Supplementary Fig. 5. Dimensionality reduction analysis of features in cancer detection. Principal component analysis (PCA) of cfDNA from healthy individuals (green) and eight cancer types: colorectal (yellow), gastric (gray), liver (orange), pancreatic (purple), lung (red), breast (pink), ovarian (navy), and prostate (blue) cancer. PCA was performed separately for four features: (a) average methylation fraction (AMF), (b) copy number variation (CNV), (c) fragment size distribution (FSD), and (d) fragment size ratio (FSR). The scatter plots display sample distributions along PC1 and PC2, with the explained variance for each principal component indicated PCA analysis were conducted using 2.4 million AMF marker regions, 294k CNV marker regions, 30 FSD markers, and 25k FSR regions.

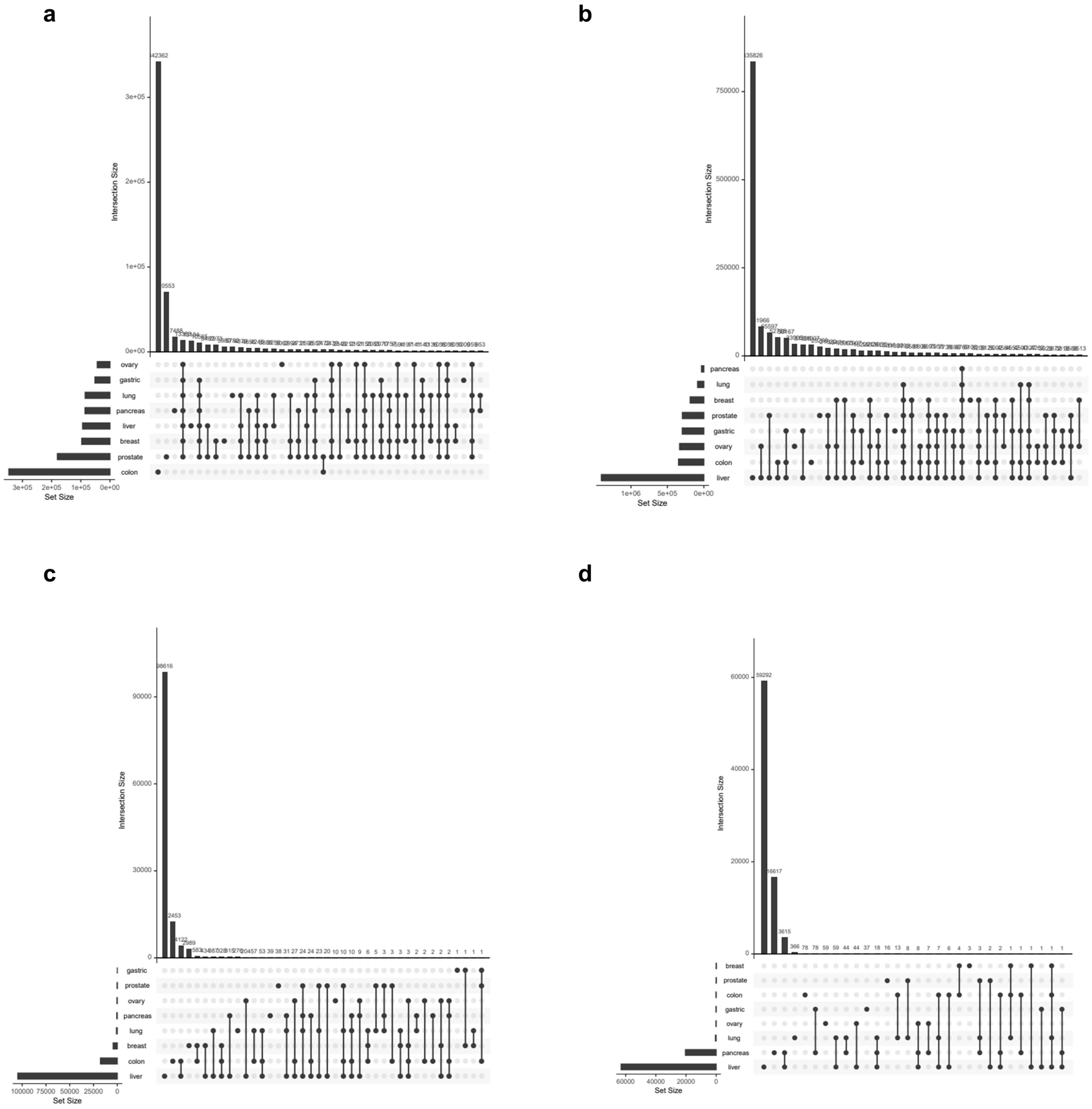

Supplementary Fig. 6. Upset plot between cancer and healthy. (a) Hyper- and (b) hypo-methylation markers between tumor tissue and healthy cfDNA. (c) Hyper- and (d) hypo-methylation markers between cancer cfDNA and healthy cfDNA

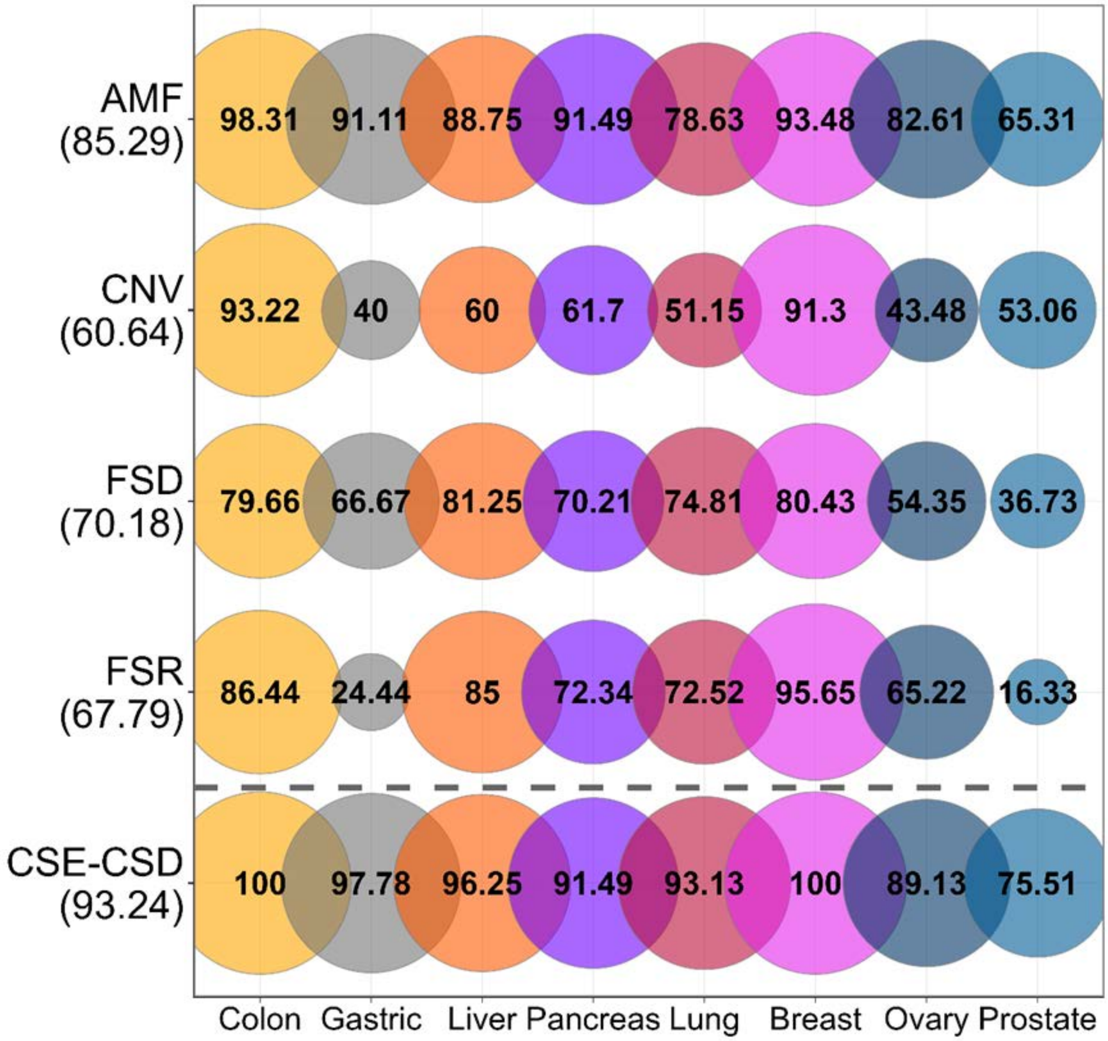

Supplementary Fig. 7. CSD Performance of individual features and CSE-CSD model across cancer types. The bubble plot shows the performance of individual features — average methylation fraction (AMF), copy number variation (CNV), fragment size ratio (FSR), and fragment size distribution (FSD) — as well as the CSE-CSD model for eight cancer types: colorectal, gastric, liver, pancreatic, lung, breast, ovarian, and prostate cancers. The size of each bubble represents the proportion of correctly classified samples for each feature and cancer type, with the percentage value displayed inside the bubble. The dashed line separates the individual features CSD from CSE-CSD model, which demonstrates the highest overall performance across cancer types.

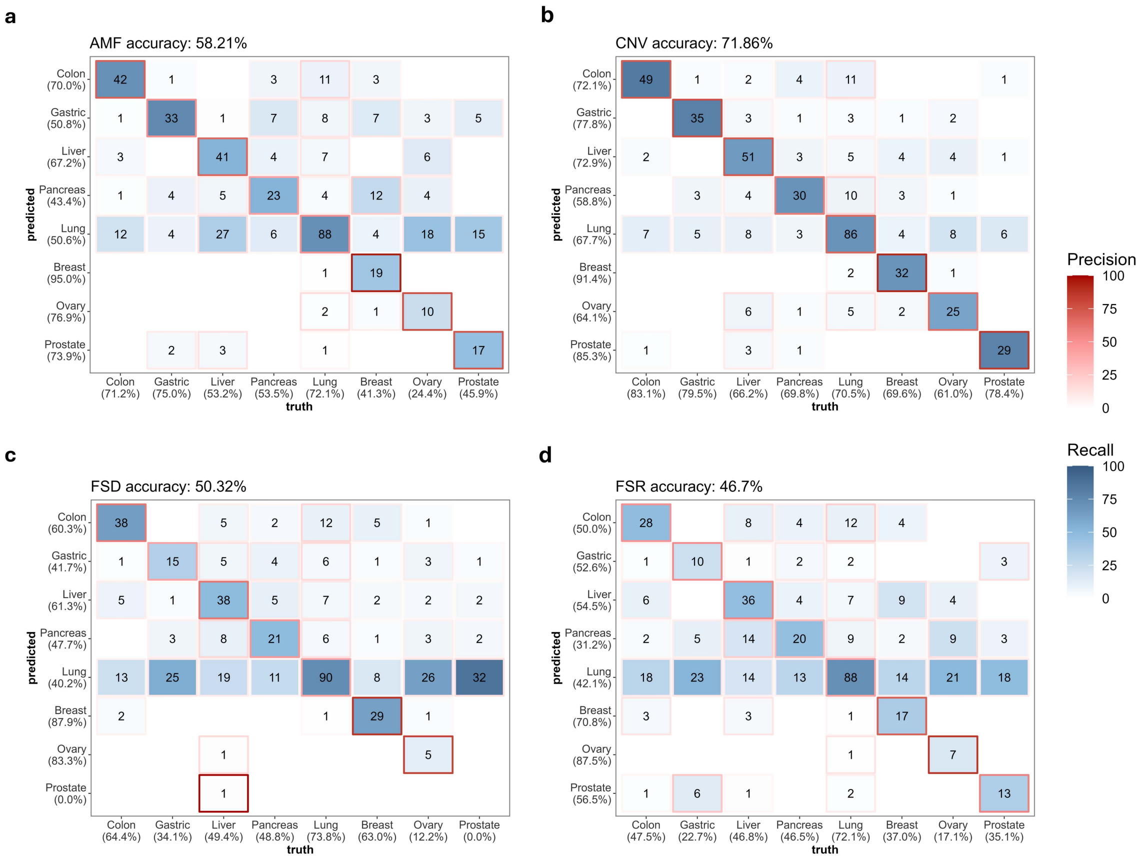

Supplementary Fig. 8. Confusion matrices for individual features across eight cancer types. Confusion matrices illustrate the classification performance of individual features — (a) average methylation fraction (AMF), (b) copy number variation (CNV), (c) fragment size distribution (FSD), and (d) fragment size ratio (FSR) — across eight cancer types: colorectal, gastric, liver, pancreatic, lung, breast, ovarian, and prostate cancers. Rows represent predicted classes, while columns represent actual cancer types. Blue gradient shading within cells indicates recall, while red gradient borders represent precision. Overall accuracy for each feature is displayed at the top of each matrix.

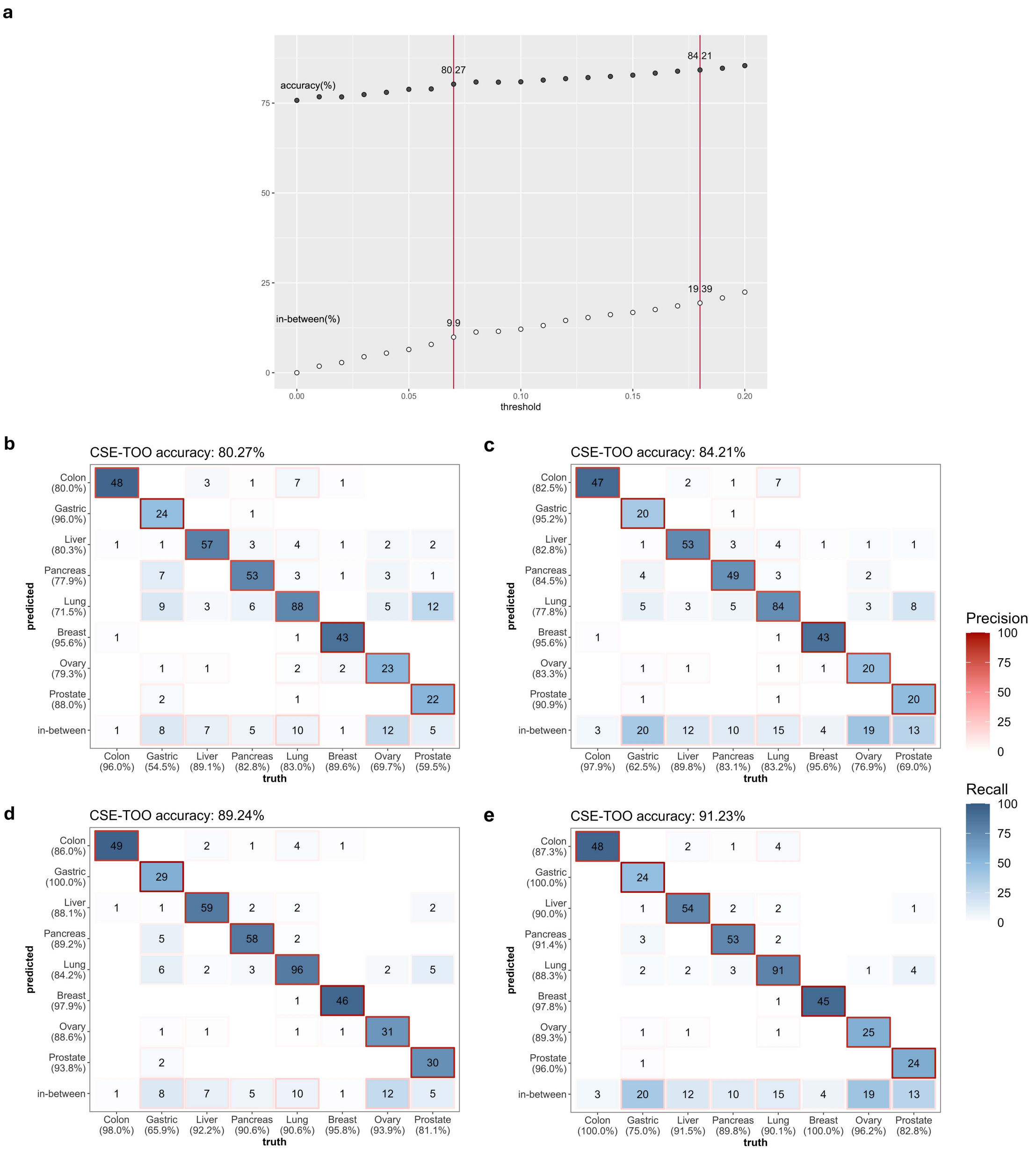

Supplementary Fig. 9. CSE-TOO prediction performance for test dataset. (a) Dot plot illustrates the determination of in-between thresholds for tissue-of-origin classification. The difference between the top 1 and top 2 probabilities was calculated, and thresholds ranging from 0 to 0.2 were applied in 0.01 increments. The plot shows the proportion of in-between classifications (open circles) and accuracy (filled circles) at each threshold. Key thresholds discussed in the study (0.06 and 0.1) are highlighted with red lines. (b-e) Top 1 and top 2 confusion matrices showing CSE-TOO prediction performance at two thresholds. The left panel represents results at a threshold of 0.05, and the right panel represents results at a threshold of 0.09. The upper matrices display top 1 predictions, while the lower matrices show top 2 predictions. The confusion matrices depict relationships between predicted classes (y-axis, including the in-between class) and actual cancer types (x-axis, eight cancer types). Blue gradient shading within cells indicates recall, while red gradient borders represent precision. Overall CSE-TOO prediction accuracy (%) is shown at the top of each matrix.

Supplementary Table 1. IRB approved number

Cancer type	Samples type	Source	Number of samples	IRB no.
Healthy	blood	SNUH	140	H-2201-128-1294
		HMF	142	HNR2022-02
	plasma	SNUH	83	H-1805-049-944
Colon	plasma	SNUH	131	H-1708-031-875
	tumor tissue	SNUH	106	
	adjacent normal tissue	SNUH	20	
Liver	plasma	AJUH	62	AJIRB-BMR-EXP-21-525
		SNUH-HUB	51	H-2201-128-1294
	tumor tissue	AJUH	30	AJIRB-BMR-EXP-21-525
		SNUH-HUB	20	H-2201-128-1294
	adjacent normal tissue	AJUH	26	AJIRB-BMR-EXP-21-525
Lung	plasma	SMC	125	2022-10-034
		SNUH	14	1907-090-1048
		SNUH-HUB	100	H-2201-128-1294
	tumor tissue	SNUH-HUB	16	H-2201-128-1294
		SNUH	15	1907-090-1048

	adjacent normal tissue	SNUH	14	1907-090-1048
Prostate	plasma	EUMC	135	SEUMC 2022-08-047-002
		SNUH-HUB	8	H-2201-128-1294
	tumor tissue	SNUH-HUB	30	H-2201-128-1294
Breast	plasma	SNUH-HUB	74	H-2201-128-1294
	tumor tissue	SNUH-HUB	50	H-2201-128-1294
Ovarian	plasma	SNUH-HUB	87	H-2201-128-1294
	tumor tissue	SNUH-HUB	34	H-2201-128-1294
Gastric	plasma	NCC	100	NCC2022-0252
	tumor tissue	NCC	32	NCC2022-0252
Pancreatic	plasma	SNUH-HUB	13	H-2201-128-1294
		SMC	87	2016-07-054
	tumor tissue	SMC	39	2016-07-054

Supplementary Table 2. Selected DMRs for colorectal, liver, and lung cancer with adjacent normal tissue

	Colorectal	Liver	Lung
**Hypo DMR**	334,670	359,800	343,626
**Hyper DMR**	12,982	2,103	12,570

Supplementary Table 3. Selected DMRs for colorectal, gastric, liver, pancreatic, lung, breast, ovarian, and prostate cancers without adjacent normal tissue

	Colon	Gastric	Liver	Pancreas	Lung	Breast	Ovary	Prostate
Hypo DMR	110,583	60,178	352,417	26,144	57,739	14,820	49,773	63,656
Hyper DMR	25,777	2,549	52,314	247,054	23,645	81,892	34,038	413,042

Supplementary Table 4. Performance comparison before and after marker selection for fixed specificity 95%

Feature	category	# of markers	Sensitivity (%) for fixed 95% specificity
AMF	before DMR selection	2,381,150 regions	84.9% (95% CI: 81.5-87.9%)
	after DMR selection	407,142 regions	84.8% (95%CI; 81.5-87.9%)
CNR	before DMR selection		42.7% (95% CI: 38.4-47.2%)
	after DMR selection		56.7% (95% CI: 52.2-61.0%)

Supplementary Table 5. Coefficients of CSE-CSD logistic regression models

Coefficients	Estimate	Std.Error	z value	Pr(>|z|)
(Intercept)	7.555505813	1.822072476	4.146654929	3.37E-05 ***
AMF	1.075521377	0.140199251	7.67137748	1.70E-14 ***
CNV	1.94633414	0.396810832	4.904942064	9.35E-07 ***
FSD	0.927692149	0.233898588	3.966215264	7.30E-05 ***
FSR	0.276350245	0.250840956	1.101695069	0.270594261

Signif. codes: 0 '***' 0.001 '**' 0.01 '*' 0.05 '.' 0.1 ' ' 1
